# Data-driven modelling and spatial complexity supports heterogeneity-based integrative management for eliminating *Simulium neavei*-transmitted river blindness

**DOI:** 10.1038/s41598-020-61194-w

**Published:** 2020-03-06

**Authors:** Edwin Michael, Morgan E. Smith, Brajendra K. Singh, Moses N. Katabarwa, Edson Byamukama, Peace Habomugisha, Thomson Lakwo, Edridah Tukahebwa, Frank O. Richards

**Affiliations:** 1 Department of Biological Sciences, University of Notre Dame, Notre Dame, IN 46556 USA; 2 The Carter Center, One Copenhill, 453 Freedom Parkway, Atlanta, GA 30307 USA; 3 The Carter Center, Uganda, 15 Bombo Road, P.O. Box, 12027 Kampala, Uganda; 4 Vector Control Division, Ministry of Health, 15 Bombo Road, P.O. Box, 1661 Kampala, Uganda

**Keywords:** Ecological epidemiology, Parasitic infection

## Abstract

Concern is emerging regarding the challenges posed by spatial complexity for modelling and managing the area-wide elimination of parasitic infections. While this has led to calls for applying heterogeneity-based approaches for addressing this complexity, questions related to spatial scale, the discovery of locally-relevant models, and its interaction with options for interrupting parasite transmission remain to be resolved. We used a data-driven modelling framework applied to infection data gathered from different monitoring sites to investigate these questions in the context of understanding the transmission dynamics and efforts to eliminate *Simulium neavei*- transmitted onchocerciasis, a macroparasitic disease that causes river blindness in Western Uganda and other regions of Africa. We demonstrate that our Bayesian-based data-model assimilation technique is able to discover onchocerciasis models that reflect local transmission conditions reliably. Key management variables such as infection breakpoints and required durations of drug interventions for achieving elimination varied spatially due to site-specific parameter constraining; however, this spatial effect was found to operate at the larger focus level, although intriguingly including vector control overcame this variability. These results show that data-driven modelling based on spatial datasets and model-data fusing methodologies will be critical to identifying both the scale-dependent models and heterogeneity-based options required for supporting the successful elimination of *S. neavei*-borne onchocerciasis.

## Introduction

In recent years, there has been growing appreciation of the role that mathematical models can play in guiding the control or eradication of the major preventable helminthic diseases, ranging from soil-transmitted helminthiases to schistosomiasis, lymphatic filariasis (LF), and onchocerciasis^1–11^. This recognition has been catalysed by the expectation that quantitative mathematical models can provide a robust scientific basis for predicting the course of infection resulting from perturbations induced by interventions^12^. A second major reason is the implicit belief that making such ecological predictions is possible, despite the complex, nonlinear, and open nature of the parasitic systems being studied and the approximations necessary to translate understanding of these systems into computer programs^4,13–15^. Despite these constraints, helminth transmission models are created and used today in the belief not only that they offer the best available tools for providing dependable predictions of the future states of such systems, but also that they will continue to evolve towards more realistic representations of parasitic systems as knowledge of transmission processes and structures improves over time^16,17^.

Apart from the above challenges, which in essence question the intrinsic predictability of any complex ecological system^15,18,19^, a growing strand of work in ecological modelling has also underlined the additional difficulties posed by spatial variability and complexity for efforts to understand and predict the dynamics of natural systems across landscapes of interest^4,20–24^. This topic is also receiving increased attention among the major agencies and actors in parasitic disease control given the emergent concerns regarding the consequences of variations in initial endemicity levels and the impacts of persisting spatial hotspots of transmission for current efforts to accomplish area-wide disease elimination^25–27^. At its core, these concerns relate to the recognition that any measured response in a location inevitably reflects the uniqueness of inputs and system characteristics of that site, and thus that any process-based model aiming to simulate such an outcome must ideally represent a balance between a generalized process paradigm and the empirical relationships that actually describe the individual processes governing a system in a particular location^28^. This impact of the unique and particulate system characteristics of a location on the parameters required by a model may be such as to necessitate the need for sets of spatially varying values for effectively constructing distributed physically-based models if reliable predictions of the dynamics of a natural system are to be made everywhere across a spatially complex domain; the corollary to this situation is clear, *viz*. that such heterogeneity will severely limit the application of an ideal, non-spatial, equilibrium model for predicting local effects, suggesting instead the need for additional testing and calibration when switching to other areas or scale^21,22,29^.

This critical sensitivity of parasite transmission processes to spatially varying factors and its effects on intervention outcomes have been highlighted by our own recent work, which has demonstrated how complex environmental and geographic heterogeneities influencing parasite transmission dynamics between communities can lead to significant variations in transmission breakpoints and hence timelines to elimination for the mosquito-borne parasitic disease, LF^25,30–32^, indicating that ignoring the uniqueness of place may lead to use of poor models for many places leading to biased results (ie. producing/accepting high false positive or Type I errors^14^) for guiding effective parasite control. While these studies have suggested the need for a heterogeneity-based paradigm for both modelling and managing a macroparasitic disease^4,25,30^, it is not clear whether the impact of spatial complexity is as significant and applies also to less locally-driven vector-borne diseases exhibiting more homogeneous transmission patterns within an endemic zone. According to this perspective, it is possible that a homogeneous spatial domain may exist within which one may assume the operation of an aggregated, stable, and stationary or constant relationship between the processes and patterns of transmission^21,22^, which would obviate the need to further address the effects of any finer scale spatial heterogeneities. Thus, in such cases, an ecological system’s behaviour is thought to be limited by both the operation of its biotic components as well as the environmental constraints imposed by higher organization levels^33^. In practice, this hierarchical principle means that coherent models may be built for the resulting constraining environmental envelops or regions without a noticeable loss in predictive power^21^.

The above also indicates that if ideal theory or a spatially homogeneous model is unable to provide reliable predictions for a particular location, then spatial data will need to be collected and assimilated effectively into models to learn the features of local dynamics, whether this be for finer or relatively coarser spatial scales. This means that model-data assimilation (also referred to as “model-data fusion” or “inverse modelling” approaches^34–37^), including Bayesian data assimilation (DA) frameworks^38,39^, by which observations are used to infer a model and quantify uncertainty, will need to play a bigger role if progress is to be made for delivering reliable predictions for different locations. This also implies that in the absence of closed-form solutions to theoretical models for addressing variable dynamics across complex particulate and contextual environments, numerical simulation coupled to spatial (and temporal) data will constitute the primary tool to investigate system behaviour^16,22^. Finally, if we find complex heterogeneous dynamics to be a major feature of parasite transmission dynamics across a landscape, then we require to recognize the limitations of a homogeneity-based management approach to parasite control and consider how scale-driven heterogeneity-based management might be implemented to optimize efficiency^40,41^.

Here, our primary goal is to extend our ongoing work on using data-driven modelling approaches to address the impact of spatial complexity for simulating the population dynamics and control of major vector-borne macroparasitic infections across an endemic domain, focussing here on the *Simulium neavei* -borne filarial disease, onchocerciasis, that causes river blindness in Africa^42^. We accomplish this by applying a Bayesian computational framework that allows the statistical integration of transmission models with human infection and vector abundance data assembled from field community surveys carried out in endemic sites, in order to identify and evaluate the predictions arising from the resulting behaviourally acceptable *S. neavei*-transmitted onchocerciasis models applicable to each setting. Community survey data from sixteen sites providing the driver and boundary condition information required for model estimation (*viz*. biting vector abundance, age-infection prevalence and intervention coverage data) assembled from across Western Uganda as part of that country’s national onchocerciasis elimination program were used in this study. We employed the location-specific models learned from each site to address several fundamental questions germane to investigating the impact of between-site heterogeneity on the dynamics of onchocerciasis elimination this study region, such as: 1) what is the significance of between-site heterogeneity on *S. neavei*-borne onchocerciasis transmission dynamics in the relatively spatially-synchronous riverine catchment or watershed environments that support its transmission?; 2) how does this spatial complexity interact with currently applied interventions for affecting parasite elimination in a site?; and 3) finally, what do the results imply for managing the area-wide elimination of onchocerciasis in these endemic regions?

## Results

### Model fits to baseline age-profiles of mf infection

**Figure 1:**
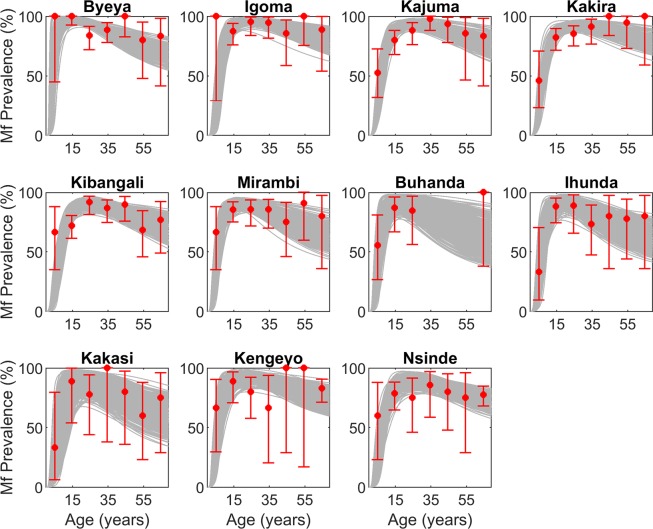
Model-estimated age profiles of microfilariae prevalence at baseline compared to observed data. The focus ABR model predictions (gray curves) generated by the SIR-selected parameter sets according to their likelihood to the site-specific age-stratified mf prevalence data at baseline (red points) are displayed for the 11 sentinel sites in the Itwara and Kashoya Kitomi foci. Error bars denote the 95% binomial confidence intervals around the observed prevalence data. Pass/fail scores (see text) indicate that there was a greater than 92% agreement between the model estimated prevalence and the observed age data for all sites except Byeya.

We calibrated our deterministic population model describing onchocericasis transmission to baseline infection data from 11 villages from two foci in Uganda (see Methods). Our model fitting procedure uses a Bayesian Melding (BM) framework in which the posterior parameter vectors are selected using the Sampling Importance Resampling (SIR) algorithm (see Methods).The predictions generated by the 500 SIR-selected models in relation to baseline infection data (age-stratified mf prevalence) from each of the 11 hyperendemic villages from Itwara and Kashoya Kitomi based on the focus-level ABRs observed in each focus (Table 1) are shown in Fig. 1. A binomial generalized additive model testing for differences in the observed age prevalences found that village as well as foci were significant predictors of the variation in these prevalences (*p*-values < 0.05), highlighting the fact that despite the selection of these sentinel villages from an apparently spatially synchronous transmission setting (at least within each focus), host infection prevalence varied both locally between villages and across transmission zones. Pass/fail scores indicate that for all sites except Byeya there was a greater than 92% agreement between the model estimated prevalence and the observed age data (Supplementary Table S1), indicating that the best-fitting focus ABR models generated in this study are in general behaviorally able to produce baseline age-infection curves reflective of the spatially-variable patterns measured in these villages.

**Table 1:** Village-specific epidemiologic and entomologic data used in this study.

Focus	Sentinel village	Black fly species	ABR (focus)	Baseline mf prevalence	Follow-up mf prevalence^a^	Vector Control^b^	MDA shift^c^ (year)
Itwara	Byeya	*Simulium neavei*	31779	91.4%	0.50%	mid 1995 –June 2003	No
	Igoma			92.1%	1.30%		
	Kajuma			85.2%	0.40%		
	Kakira			86.0%	—		
	Kibangali			81.5%	—		
	Mirambi			84.2%	—		
Kashoya Kitomi	Buhanda	*Simulium neavei*	28416	81.3%	—	Jul 2007 –Oct 2010	Yes (2007)
	Ihunda			81.0%	4.40%		
	Kakasi			76.3%	—		
	Kengeyo			83.1%	3.20%		
	Nsinde			77.9%	4.30%		
Bwindi	Muko	*Simulium neavei*	—	50.0%	0.0%	Jan 2015 – present	Yes (2007)
	Kashaka			54.0%	5.9%		
	Mugombwa			60.0%	4.3%		
	Kikobero			84.0%	6.2%		
	Suma			18.0%	4.9%		

- indicates no data available

^a^Mf prevalence surveyed in 2004 in Itwara and Kashoya Kitomi, 2005 in Bwindi

^b^the period during which vector control (larviciding) was implemented

^c^indicates whether there was an MDA policy shift from annual to biannual treatments.

### Estimation of site-specific annual biting rates

**Figure 2:**
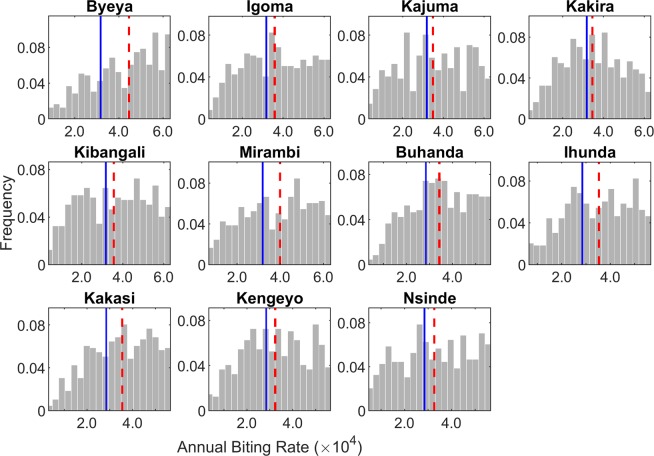
Model-estimated site-specific annual biting rates. The histograms show the relative frequencies of the model-estimated annual biting rates for each village studied in the Itwara and Kashoya Kitomi foci. Wilcoxon signed rank test results indicate that the distributions of estimated site-specific ABRs do not come from distributions whose median is equal to the focus-level ABR (blue solid line), suggesting a significant difference in the two values (*p*-values < 0.05 for all sites). The estimated site-specific ABR distributions between sites are statistically significantly different as given by Kruskal-Wallis tests comparing ABRs between all sites as well as between sites within each focus (*p*-values < 0.05).

**Figure 3:**
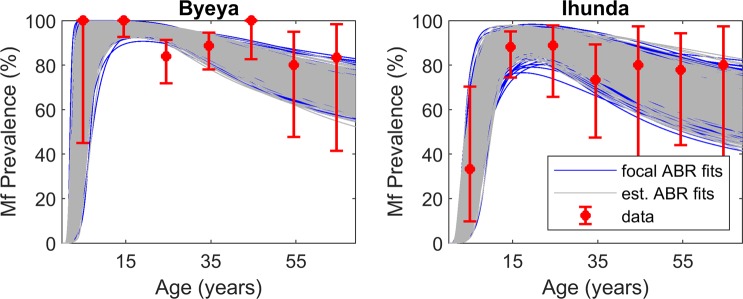
Comparison of baseline model fits from the focus ABR versus site-specific ABR models. The baseline age-prevalence curves generated via model simulations using estimated site-specific ABR (gray curves) represent the village-level infection age-profiles similarly to those of model simulations carried out using focus-level ABRs (blue curves). Model estimates given either ABR inputs captured the observed data well (Supplementary Table S2). Red data points represent the age-specific prevalence data with 95% binomial confidence intervals.

The sites studied here from the Itwara and Kashoya Kitomi foci had only focus-level ABR data available to guide the selection of onchocerciasis models applicable to each village setting (Table 1); this raised the question as to whether using the focus ABR as a driving variable allows the appropriate identification of local (village level) models. We investigated this question by reverse engineering the expected site-specific ABR values given the observed age-infection prevalence data (see Methods), and comparing these model predictions with the measured focus-level ABR values. The model-generated distributions of site-specific ABRs are shown in Fig. 2, and Wilcoxon signed rank tests suggest that these varied from the corresponding focus ABR values in each site (*p*-values < 0.05). Additionally, the estimated site-specific ABR distributions were also statistically significantly different between all sites, and within each of the two foci (Kruskal-Wallis tests comparing the ABRs of sites (*p*-values < 0.05) in each case, respectively). However, the consequence of this difference was found not to be appreciable with respect to the SIR models identified as best-fitting the present data, given that 1) the site-specific ABR models captured the observed baseline infection data similarly to the focus ABR models (Fig. 3), and 2) comparison using the relative root-mean-square error (*ReRMSE*) metric confirming that the performance of the site-specific ABR models did not differ quantitatively from that of focus ABR models (Supplementary Table S2). These results demonstrate, firstly, that using focus-level ABR values, at least from the present relatively homogeneous onchocerciasis endemic zones, may be sufficient to model local transmission differences, and, secondly, that if baseline ABR information is missing, as is often the case with national field surveys, model-estimated site-specific ABRs can reliably facilitate the learning of local behaviourally suitable onchocerciasis models.

### Assessment of posterior parameter distributions

The BM-based data assimilation approach basically facilitates the learning of local onchocerciasis models by updating parameter estimates from their original uniform priors using the available locality-specific mf prevalence and ABR data. The prior and posterior parameter distributions from the best-fit SIR focus ABR models for each site were compared by Kolmogorov-Smirnov tests, and 19 of 22 parameter distributions tested were found to have been significantly changed by the Bayesian updating procedure (*p*-values < 0.05, Table 2). The corresponding results for the site-specific ABR models for sites in Itwara and Kashoya Kitomi are given in Supplementary Table S3 and do not show a notable difference compared to the focus models, suggesting: 1) that the local data were informative for updating posteriors, and 2) that the use of focus-level ABR was sufficient for calibrating the present models to reflect the local transmission conditions obtaining in the investigated sentinel monitoring sites.

**Table 2:** Kolmogorov-Smirnov results comparing prior and posterior parameter distributions in each village given the focus-level ABR.

Sentinel village	Model parameter^b,c^																					
	H_b_	g	α	^b^k_0_	k_Lin_	^b^κ	r	σ	ψ_1_	ψ_2_	μ_w_	γ	b	c	H_Lin_	I_C_	S_C_	I_Cmin_	^b^σ_L_	τ	δ	σ_e_
Byeya	1^a^	1	1	1	1	0	1	0	1	1	1	0	1	1	1	1	1	1	0	1	1	1
Igoma	1	1	1	0	1	1	1	0	1	1	1	0	1	1	1	1	1	1	0	0	0	1
Kajuma	1	1	1	1	1	1	1	1	1	1	1	1	1	1	1	1	1	1	1	1	1	1
Kakira	1	1	1	1	1	1	1	1	1	1	1	1	1	1	1	1	1	1	1	1	1	1
Kibangali	1	1	1	0	1	0	0	1	1	1	1	1	1	1	1	1	1	1	0	1	0	1
Mirambi	1	1	1	0	1	0	1	1	1	1	0	1	1	1	1	1	1	1	0	0	1	1
Buhanda	1	1	1	0	1	0	0	1	1	1	0	1	1	0	1	1	1	1	0	0	0	1
Ihunda	1	1	1	0	1	0	0	0	1	1	0	0	1	0	1	1	1	1	0	1	0	1
Kakasi	1	1	1	0	1	0	0	0	1	1	0	0	1	1	1	1	1	1	0	0	0	1
Kengeyo	1	1	1	1	1	1	1	1	1	1	1	1	1	1	1	1	1	1	1	1	1	1
Nsinde	1	1	1	1	1	0	0	1	1	1	1	1	1	1	1	1	1	1	0	0	1	1

^a^Parameters whose posterior distributions differed statistically significantly from their prior distributions (*p*-value < 0.05) according to the focus ABR models are identified with a 1, while those posteriors which did not differ from their priors are identified with a 0.

^b^Parameters did not differ from their priors for a majority (>5) of the sentinel villages.

^c^See Supplementary Table S8 for parameter definitions.

Kolmogorov-Smirnov tests comparing posterior parameter distributions between site-specific and focus ABR models, however, showed that the distributions for five parameters are significantly different, indicating their sensitivity to the choice of ABR type (whether focus or site-specific) used in the present simulations (Table 3). Interestingly, most of the parameters that are affected by this sensitivity to the specific ABR used are those related to exposure (*H*_*b*_, *ψ*_1_, *ψ*_2_, *H*_*Lin*_), suggesting that these ecological parameters can compensate for the change in ABR values employed in order to reproduce the observed mf infection profiles. Note the fact that such different parameter and ABR combinations are able to reproduce the observed age infection profiles in a site equally well indicates that complex interactions and trade-offs exist between parameters or that multiple model representations^20,43,44^ can act to provide acceptable simulations for the onchocerciasis transmission system. This further emphasizes the importance of explicitly allowing for the effects of parameter uncertainty in the modelling of this disease.

**Table 3:** Kolmogorov-Smirnov results for comparing posterior parameter distributions from the focus and site-specific ABR models.

Sentinel village	Model parameter^a,c^																					
	H_b_	g^a^	α^a^	k_0_	^a^k_Lin_	κ^a^	r^a^	σ^a^	ψ_1_	ψ_2_	^a^μ_w_	γ^a^	b^a^	c^a^	H_Lin_	^a^I_C_	^a^S_C_	^a^I_Cmin_	^a^σ_L_	τ^a^	δ^a^	^a^σ_e_
Byeya	1^b^	0	0	0	1	1	0	1	0	1	0	0	0	1	1	0	1	0	0	0	0	0
Igoma	1	1	0	0	1	0	0	0	1	0	0	1	1	0	1	0	0	0	0	0	0	0
Kajuma	1	1	1	1	1	1	1	1	1	1	1	1	1	1	0	0	1	0	0	0	0	0
Kakira	0	1	1	1	1	1	1	1	1	1	1	1	1	1	1	1	1	0	0	0	0	0
Kibangali	1	1	0	1	1	0	0	0	1	1	1	1	0	0	1	0	0	0	0	0	0	0
Mirambi	1	0	1	1	0	0	0	1	1	1	0	0	0	0	0	0	0	0	0	0	0	0
Buhanda	1	0	0	1	0	0	0	0	0	0	0	0	0	0	0	0	0	0	0	0	0	0
Ihunda	1	0	0	0	0	0	0	0	0	0	0	0	0	0	1	0	0	0	0	0	0	0
Kakasi	0	0	0	0	0	0	0	0	0	0	0	0	0	0	1	0	0	0	0	0	0	0
Kengeyo	0	0	1	0	0	0	1	0	0	0	1	1	1	1	1	0	0	0	0	0	0	0
Nsinde	1	1	1	1	0	0	0	0	1	1	0	0	1	0	0	1	0	0	0	0	0	0

^a^Parameters did not differ between models across a majority (>5) of the sentinel villages.

^b^Parameters whose posterior distributions statistically significantly differed from their prior distributions (*p*-value < 0.05) according to the focus ABR models are identified with a 1, while those posteriors which did not differ from their priors are identified with a 0.

^c^See Supplementary Table S8 for parameter definitions.

**Figure 4:**
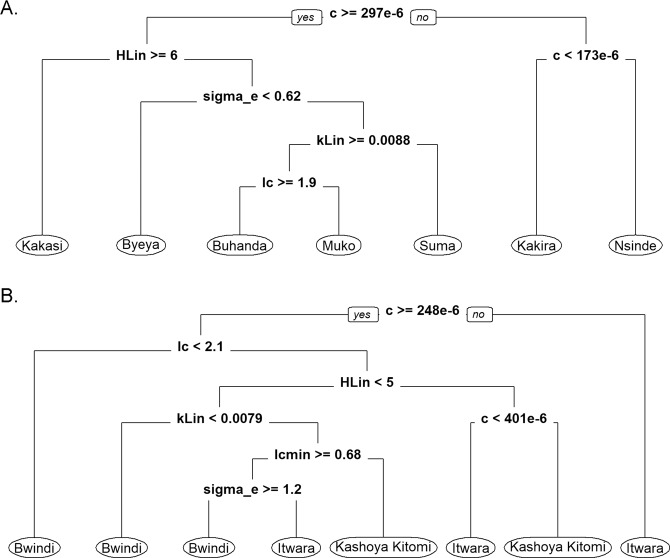
Classification tree highlighting model parameters underlying differences between sentinel sites (**A**) and foci (**B**). The best-fitting parameter sets from each of the sites from the Itwara, Kashoya Kitomi, and Bwindi foci for the site-specific ABR models were analyzed here. Village and focus-level models were classified according to their fitted parameter values using the *rpart* package in R. The trees pruned with the complexity parameter set to 0.005 and a tree depth of 6 are shown here. The top five variables by importance were *c, H*_*Lin*_, *σ*_*e*_, *K*_*Lin*_ and *I*_*c*_ in the case of the village-specific models, whereas these were *c*, *I*_*c*_, *H*_*Lin*_, *K*_*Lin*_, *I*_*cmin*_, and *σ*_*e*_ in the case of models aggregated at the focus level.

We used the posterior parameter estimates from each of the present sites next to investigate which parameters most strongly contributed to differences in between-site and –foci transmission using classification tree analysis^25,30^. For assessing between-site variation in local model parameters, the whole set of data from all 16 sites were used to perform the analysis given that carrying out separate between-site analyses for each focus were precluded by the relatively small number of villages sampled per focus. The resulting classification tree from the application of the site-specific ABR models truncated to show only the first few nodes (tree depth of 6) is portrayed in Fig. 4A, and together with the tree obtained using the focus ABR models (Supplementary Fig. S1), indicate that the only consistent parameters found to be most important for underlying the observed between-site transmission heterogeneity are related to host immune response (*c*, and *Ic*), exposure (*H*_*Lin*_), infection aggregation (*K*_*Lin*_) and excess vector mortality due to mf infection (*σ*_*e*_). The corresponding results for the between-foci models (model parameters aggregated across sites within a focus) are shown in Fig. 4B, and these demonstrate a clear separation in the parameter values applicable to the Itwara and Kayshoya Kitomi foci in comparison with the Bwindi focus. Interestingly, the parameters that differentiated the estimated models between these foci again included the *I*_*c*_, *K*_*Lin*_ and *σ*_*e*_ parameters (Fig. 4B), with values apparently related to differences in worm prevalence or density in the case of *I*_*c*_ (lower values for Bwindi sites which also exhibited lower baseline mf prevalences) and infection aggregation (higher aggregation of infection in Bwindi ( = lower *K*_*Lin*_ values)). The latter result (higher infection aggregation) may also underpin the excess vector mortality estimated for this focus presumably due to a greater proportion of individuals with relatively higher intensities of infection living in this focus.

### Model fits to baseline mf infection in the absence of ABR data

**Figure 5:**
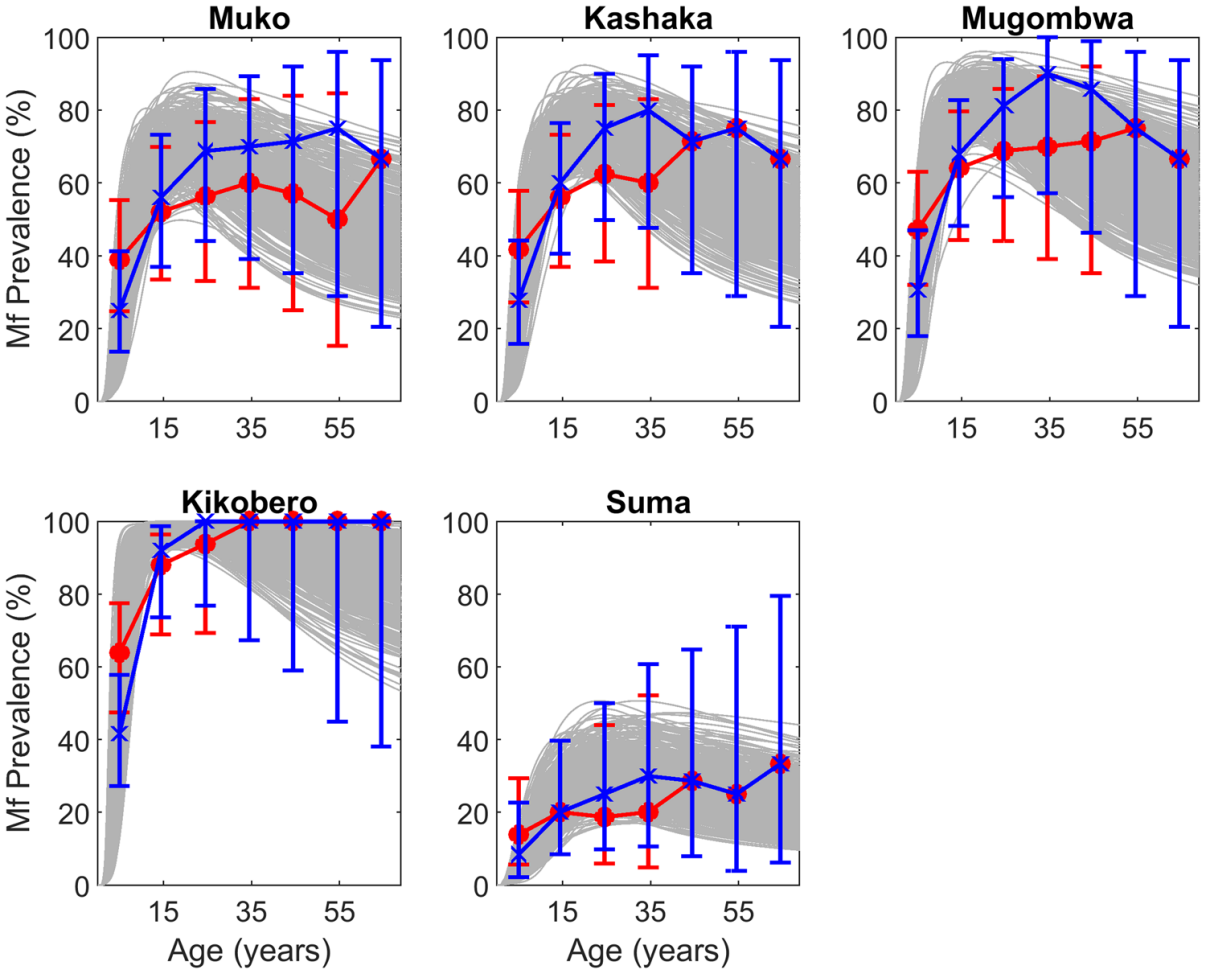
Model-estimated age profiles of microfilariae prevalence at baseline compared to observed data in Bwindi sites. Because the baseline prevalence data for these sites were aggregated for the entire population, age-stratified prevalence was estimated for two major onchocerciasis age-infection patterns – plateau (red points) and convex (blue crosses). The model curves generated by the SIR-selected parameter sets according to their likelihood to the estimated mf prevalence profiles are shown in gray. Error bars denote the 95% binomial confidence intervals around the predicted infection prevalence points for a conservative sample size of 100. Because no ABR information was available for this focus, the model-estimated ABR for each site was used to simulate the prevalence data (Supplementary Fig. S2).

As noted in Methods, we used data from five sentinel villages in the hypo- and mesoendemic Bwindi focus in order to introduce greater variability in the types of endemic settings modelled in this study (Table 1). However, as there were no data on ABR available from any of these villages, we needed to apply the site-specific ABR estimation technique described above in order to identify the local models for these villages. We also needed to estimate the age-prevalence curves from the available overall community baseline mf prevalences in these sites (see Methods). The resulting SIR model fits to these estimated age-infection data are depicted in Fig. 5, and indicate that using these approximations can allow discovery of reasonably good models for these sites. The corresponding site-specific ABR distributions estimated by these respective village-specific models are displayed in Supplementary Fig. S2. Note results from these sites were only included in subsequent analyses related to simulations using site-specific ABR models.

### Mf prevalence breakpoints

Mf breakpoints corresponding to both the ABRs and the threshold biting rates (TBRs) were calculated for the desired elimination probability (EP) of 95% (Table 4). These calculations were performed using the best-fitting parameter vectors for the baseline prevalence data for both the focus and site-specific ABR models. In the majority of cases, the breakpoint distributions generated by the site-specific ABR models did not statistically differ from those generated by the focus ABR models (Kruskal-Wallis tests, Supplementary Table S4), further suggesting that focus ABR models are sufficiently able to capture local transmission characteristics in relatively homogeneous transmission zones. Kruskal-Wallis tests show that the mf breakpoints and TBRs differed significantly (*p*-values < 0.05) between sentinel villages and between foci. However, the numerical values of the mf breakpoint prevalences given in Table 4 show that these difference may not be large enough to be practically meaningful. Additionally, these critical infection thresholds, irrespective of location, are low-valued (much lower than the WHO suggested threshold value of 1% mf prevalence^45^) and are found to be significantly much higher at TBR than at ABR.

**Table 4:** Model-derived mf breakpoint values at ABR and TBR given the focus and site-specific ABR models.

Sentinel village	Focus ABR	Site-specific ABR (95% CI)	Baseline mf prevalence	Focus ABR models		Site-specific ABR models	
				At TBR (% mf)	At ABR (% mf)	At TBR (% mf)	At ABR (% mf)
Byeya	31779	44608 (17063–62287)	91.40%	1.8820	0.0976	1.3900	0.0699
Igoma	31779	35872 (10357–60948)	92.10%	1.6569	0.0932	1.9459	0.0694
Kajuma	31779	34806 (8642–59979)	85.20%	1.8038	0.0825	1.6062	0.0739
Kakira	31779	34568 (11060–59092)	86.00%	1.4432	0.0915	1.9469	0.0822
Kibangali	31779	35667 (9442–60439)	81.50%	1.2193	0.0840	1.6423	0.0711
Mirambi	31779	39857 (13109–60590)	84.20%	1.2810	0.0676	1.9136	0.0712
Buhanda	28416	34254 (11585–54434)	81.30%	1.1391	0.0914	1.3779	0.0830
Ihunda	28416	35260 (12843–53979)	81.00%	1.2955	0.1039	1.5533	0.0714
Kakasi	28416	35392 (12208–54354)	76.30%	1.5198	0.1038	1.3843	0.0855
Kengeyo	28416	32336 (9333–53862)	83.10%	1.3909	0.0796	1.8344	0.0868
Nsinde	28416	32615 (8763–54416)	77.90%	1.7744	0.1010	1.4551	0.0766
Muko	N/A	29917 (9414–47766)	50.0%	—	—	2.4183	0.0856
Kashaka	N/A	28415 (9471–47945)	54.0%	—	—	1.0683	0.0907
Mugombwa	N/A	29690 (9734–47718)	60.0%	—	—	1.3340	0.0986
Kikobero	N/A	27878 (9438–46539)	84.0%	—	—	1.2919	0.0848
Suma	N/A	25668 (8041–47376)	18.0%	—	—	1.9317	0.0938

The mf breakpoint values shown were derived from the site-specific breakpoint distributions to represent a 95% elimination probability.

### Impact of between-site heterogeneity on onchocerciasis elimination

An array of intervention strategies were simulated to evaluate the impact of spatial variability and complexity in transmission on timelines to crossing the estimated elimination threshold values in each site. The control measures simulated consist of different combinations of annual or biannual MDA treatments with ivermectin, the presence or absence of vector control, and population drug coverage of 40–100% for up to 50 years. For modelling the effects of larvicidical (Abate) application, we first used data on observed changes in the number of flies over time as a result of applying this control measure to riverine habitats – data obtained from sites in the Kashoya Kitomi focus – in order to estimate the functional form describing the expected rate of temporal decrease in black fly biting density due to this vector control strategy. An exponential decay function best described the observed decrease in fly numbers giving a value of *A*_0_ = 0.44 (Supplementary Fig. S3), and we applied this function to model the impacts of larvicide assuming that the application and temporal effects were similar in all three study regions.

**Figure 6:**
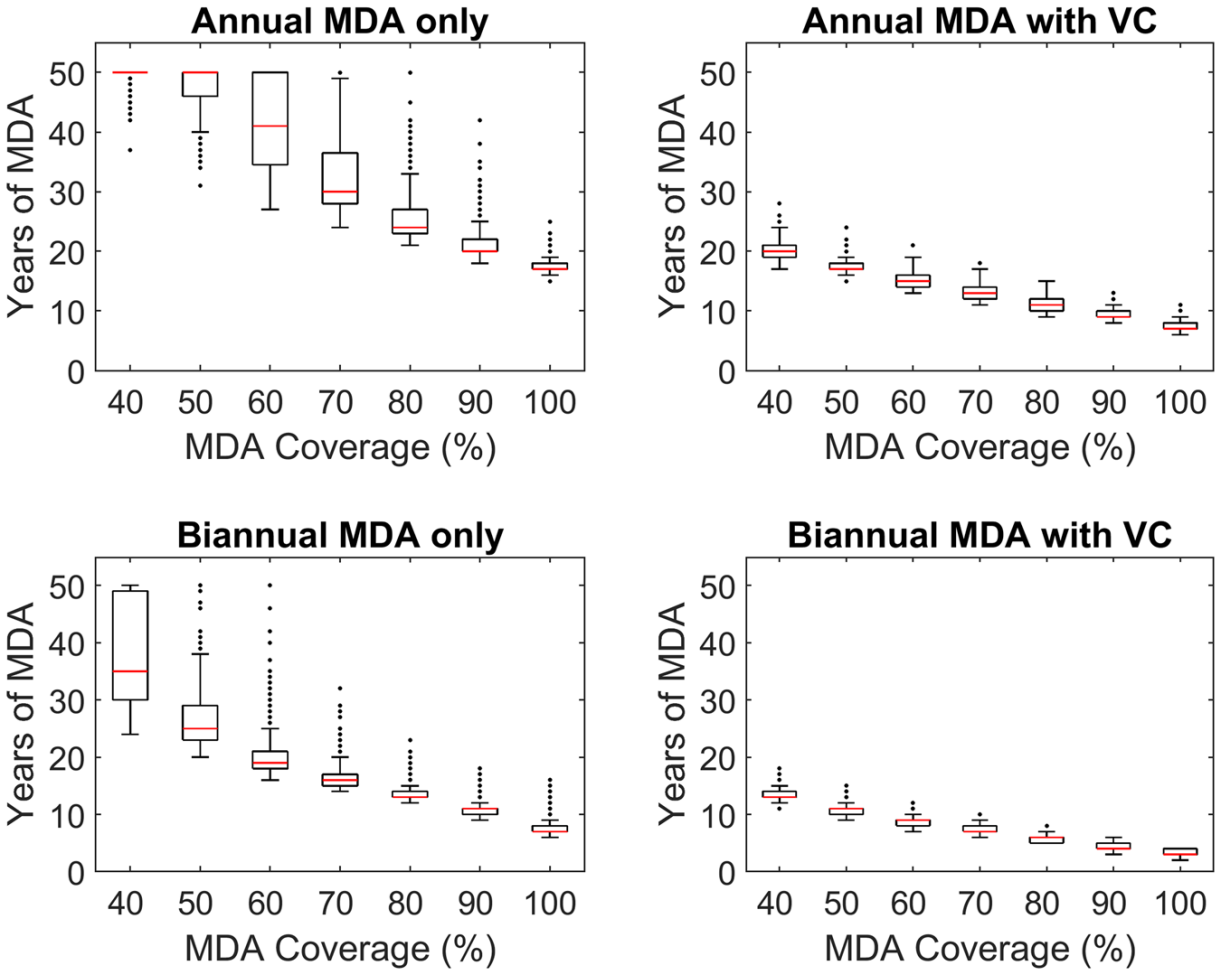
Years of MDA required for Byeya under different control strategies. Timelines refer to the number of years required to achieve the site-specific 95% elimination probability threshold (Table 4). Vector control applied in these scenarios assumes larviciding was implemented from the start of interventions. The results shown were generated by the site-specific ABR models for Byeya.

Simulation results on the effectiveness of the four MDA-based strategies in terms of the number of years to reach the estimated village-specific 95% EP threshold for one sentinel site in the Itwara focus, Byeya, are shown in Fig. 6. Intuitively, and as shown, the most effective intervention scenario is high coverage biannual MDA with VC while the least effective intervention scenario is low coverage annual MDA without VC. The number of years of MDA required to reach the estimated thresholds for each sentinel site are further tabulated in Table 5 for the optimal scenario of achieving 80% MDA coverage, implementing VC, and targeting a stringent threshold (95% EP). Under this strategy, biannual MDA treatments and the use of VC can save many years of interventions over the less intensive annual MDA without VC strategy (Table 5). For example, 26 years of annual MDA without VC are required to reach the 95% EP threshold in Byeya, while only 6 years of biannual MDA with supplemental VC are required, thus potentially saving 20 years of control activities in this site. Kruskal-Wallis tests indicated that the number of years of interventions required varied significantly between foci and between villages (Supplementary Table S5), highlighting the importance of considering local transmission dynamics for planning control activities. The distributions of timelines in each site were also found to statistically vary between the focus and site-specific ABR models (Supplementary Table S6); however, as observed for the breakpoint results, the values reported in Table 5 suggest that these differences may not be large enough to be practically meaningful.

**Table 5:** Years of annual or biannual MDA (80% coverage) required with or without vector control.

Sentinel village	Focus ABR	Site- specific ABR	Baseline mf prevalence	Focus ABR models				Site-specific ABR models			
				Annual MDA		Biannual MDA		Annual MDA		Biannual MDA	
				No VC	VC	No VC	VC	No VC	VC	No VC	VC
Byeya	31779	44608	91.40%	26 (21–50)	10 (9–12)	13 (11–17)	5 (4–6)	26 (21–50)	11 (10–13)	13 (12–17)	6 (5–7)
Igoma	31779	35872	92.10%	22 (19–28)	10 (7–12)	12 (10–14)	5 (4–6)	25 (21–37)	10 (9–11)	13 (12–14)	5 (4–6)
Kajuma	31779	34806	85.20%	23 (19–27)	9 (7–11)	12 (10–13)	4 (3–6)	24 (20–32)	10 (9–11)	12 (11–14)	5 (4–6)
Kakira	31779	34568	86.00%	23 (19–32)	10 (8–12)	12 (10–14)	5 (4–6)	23 (20–30)	9 (8–10)	12 (11–14)	5 (4–5)
Kibangali	31779	35667	81.50%	22 (18–32)	10 (8–13)	12 (10–14)	5 (4–7)	23 (20–29)	9 (8–11)	12 (11–14)	5 (4–6)
Mirambi	31779	39857	84.20%	23 (19–31)	10 (8–13)	12 (11–14)	5 (4–7)	24 (20–36)	9 (8–10)	12 (11–15)	5 (4–5)
Buhanda	28416	34254	81.30%	22 (18–28)	11 (8–13)	12 (10–13)	6 (4–7)	23 (20–30)	10 (9–12)	12 (11–14)	5 (4–6)
Ihunda	28416	35260	81.00%	21 (18–27)	10 (8–13)	11 (10–13)	5 (4–7)	23 (20–29)	10 (8–11)	12 (11–14)	5 (4–6)
Kakasi	28416	35392	76.30%	21 (17–25)	9 (7–12)	11 (9–13)	5 (3–6)	22 (19–27)	10 (8–12)	12 (11–13)	5 (4–6)
Kengeyo	28416	32336	83.10%	23 (18–36)	10 (8–12)	12 (10–14)	5 (4–6)	23 (19–34)	9 (8–10)	12 (11–14)	5 (4–5)
Nsinde	28416	32615	77.90%	21 (18–27)	9 (7–10)	11 (10–13)	4 (3–5)	23 (19–29)	10 (8–11)	12 (11–14)	5 (4–6)
Muko	N/A	29917	50.0%	—	—	—	—	20 (17–25)	9 (7–11)	11 (9–12)	5 (3–6)
Kashaka	N/A	28415	54.0%	—	—	—	—	20 (17–26)	9 (8–12)	11 (10–13)	5 (4–6)
Mugombwa	N/A	29690	60.0%	—	—	—	—	21 (18–26)	10 (8–12)	11 (10–13)	5 (4–6)
Kikobero	N/A	27878	84.0%	—	—	—	—	22 (20–28)	10 (9–11)	12 (11–13)	5 (4–6)
Suma	N/A	25668	18.0%	—	—	—	—	15 (13–20)	8 (5–11)	8 (7–10)	4 (2–6)
Mean (95% CI)				22 (18–30)	10 (7–12)	12 (10–14)	5 (4–7)	22 (15–31)	10 (7–12)	12 (8–14)	5 (3–6)

The number of years represents the mean time to cross the site-specific 95% EP threshold.

An important finding highlighted by these results is that including VC using Abate applications can not only reduce the mean durations required to reach elimination, but also, strikingly, both the within and between-site variance in the predicted elimination timelines compared to the corresponding MDA only strategies. This finding suggests that including VC into MDA programs has the additional advantage that it can significantly overcome the inherent between-site heterogeneity in parasitic transmission dynamics besides improving predictive reliability. Post-hoc multiple comparison testing using the Nemenyi-test for multiple comparisons of (mean) rank of sums indicated that while the predicted timelines to elimination differed significantly between each pair of foci studied here for the MDA-only based strategies (*p*-values < 0.0001), the mean durations were reduced significantly in the Bwindi sites in comparison to both the Itwara and Kashoya Kitomi sites (*p*-values < 0.0001) - but not between the latter sites - when vector control was included in the intervention simulations (*p*-values > 0.50). (Table 5). Again, however, the practical significance of this reduction in intervention durations appeared small. These findings underscore that while transmission factors at the focus level can have significantly differential, albeit practically small, outcomes for MDA only interventions, the inclusion of vector control can overcome and reduce this variability appreciably between foci or strata even when they vary in their baseline endemicity.

## Discussion

Our central goal in this paper was to assess the impacts of spatial complexity and heterogeneity for modelling *S. neavei*-transmitted onchocerciasis and for managing the elimination of this important macroparasitic infection in typical endemic regions. We show that while spatial complexity can play a significant role in the transmission and elimination dynamics of the onchocerciasis transmitted by *S. neavei* black flies, these effects appear to operate at the scale of the larger transmission focus rather than, as for the mosquito-borne sister filarial disease, LF, at finer village-level point spatial scales^25,30,31^. Such partitioning or constraining of transmission dynamics by higher spatial levels means, as we have demonstrated here, that models can be discovered efficiently for these transmission zones by calibration to data from a few sentinel sites that capture the dominant features of transmission dynamics in such regions (although see caveats below). If applicable, this clearly enhances the efficiency and power of modelling such spatially stratified parasitic systems since it obviates the need to consider the effects of spatial (and temporal) complexity across the full range of spatial/temporal scales down to the lowest levels (ie. cell-by-cell or village point scales)^21,22,46^. We also show that such zonal or stratified partitioning of *S. neavei* -onchocerciasis transmission dynamics means that although between-site variability will still occur, this will have little practical effects on the dominant dynamics of extinction (elimination breakpoints, timelines to extinction due to interventions) within a focus or between transmission foci exhibiting relatively similar pre-control endemicity or infection patterns (Tables 4 and 5). This indicates that, depending on the intervention option (see below), outcomes of interventions are likely to vary appreciably only between transmission foci or regions that differ substantially in endemicity levels for *S. neavei-*onchocerciasis (eg. between the mesoendemic Bwindi focus compared to the hyperendemic Itwara and Kashoya Kitomi foci). Such a spatially stratified structure in the dominant transmission dynamics within an endemic zone suggests that management of *S. neavei* –transmitted onchocerciasis elimination will need to be sensitive to variable parasite transmission and extinction dynamics at the focus level rather than be based on a paradigm of uniformity that ignores this spatial heterogeneity.

The above conclusions are clearly conditioned on the ability of our modelling framework to reliably capture the dominant transmission dynamics of *S. neavei* - transmitted onchocerciasis in an endemic focus. While our BM-based modelling algorithm combines the advantageous features of mechanistic and statistical approaches to improve the estimation of local models for facilitating forecasts of interventions applied under a variety of field conditions, it is dependent, as for any data-driven predictive system, on the model structure employed, estimation procedure, and on the data used for facilitating model discovery^16,17^. Although our BM framework primarily focused attention on addressing parameter uncertainty with data, we note here firstly that the present model is based on previously established population models of onchocerciasis transmission^7,47^, with appropriate structural extensions made with regard to population-averaged mf uptake and larval development in the *S. neavei* vector host as well as the operation of different forms of host immunity in populations^48^. Furthermore, we have also secondly included all previously suggested density-dependent functions that are thought to govern onchocerciasis transmission but do not make any *a priori* assumptions concerning their occurrence using data, instead, to determine the operation of these functions in a site, which allows for a degree of updating for model structures applicable to a particular setting.

By contrast to conventional model calibration practices (which principally focus on adjustment of parameters until discrepancy between model outputs and observed data is minimized), the Bayesian basis of our Monte-Carlo BM-based model estimation technique, as we have demonstrated previously^25,30,31,49^, is also more informative given that it not only updates our knowledge of model parameters (and structure) for a site but also facilitates predictions along with associated uncertainties for modelled output variables^38,50^. In addition, it is also vitally premised on the expectation that obtaining a single, optimal, set of acceptable parameters for describing a complex multi-parameter ecological system is problematic, as parameter equifinality means that often several behavioural sets can fit observations^20,29,38^. Such model equifinality implies that there may well be many combinations of initial conditions, model behaviours and parameter sets that are consistent with the limited observations available about a particular ecological system, and that it is important to capture this complexity through selection of sets of behaviourally acceptable models, such as the present SIR-selected models, for ensuring the making of reliable predictions.

Another key component of our modelling approach is the use of site-specific data from the three transmission foci investigated in order to determine within- and between-focus transmission heterogeneity. A special feature of the site-specific mf prevalence (and ABR) data used in this analysis, however, is that these were collected in sentinel communities in locations close to riverine systems suitable for supporting *S. neavei* populations to ensure that conditions of maximal transmission are monitored in each focus. Thus, while this selection does not allow study of the full range of transmission heterogeneity and complexity likely to occur within the present onchocerciasis foci, it does allow comparison of parasite transmission intensity and variability occurring within the maximal or the dominant transmission tracts within and between each of the present endemic foci. This implies that conclusions regarding focus-level heterogeneity made in this paper should strictly be considered as alluding to the spatial variability observed for the models and their predictions applicable in these comparable maximal riverine transmission tracts within each of the investigated foci. Such simulations of worst-case settings are, however, in alignment with another implicit practical aim of our modelling goal here, *viz*. that we are able to provide predictions/forecasts relevant to supporting managerial decision making regarding the maximal durations of interventions required for achieving the interruption of *S. neavei* – borne *Onchocerca* transmission in a focus.

The values of mf breakpoints that give rise to a 95% probability of interrupting sustainable parasite transmission once breached (Table 4) represent the first such estimates produced for onchocerciasis. Previous work has largely focused on estimation of threshold vector biting rates^51–53^, although Duerr *et al*.^51^ also investigated mf breakpoint shifts in terms of the application of CDTi. Our work here has systematically extended these previous studies by addressing the values of both the mf breakpoint in the human population as well as the vector threshold biting rate that occur together in a site using data-fitted models. The results demonstrate, for the first time, not only that these critical thresholds governing breakage of *S. neavei-*borne onchocerciasis transmission are typically lower-valued (between approximately 0.06% to as relatively high as 0.10%) than the WHO suggested global threshold value of 1% mf prevalence in the presently studied sites^45^, but also that they can vary significantly both between sites and between foci. However, an important finding is that the values of these infection breakpoints within sites were found to be strikingly higher at the modelled vector threshold biting rate (TBR) with estimated values universally higher ( > at least 1.13%) than the 1% mf prevalence elimination threshold set by WHO, and, in some settings, even reaching values close to 2% mf prevalence (Table 4). This finding corroborates the results obtained previously both for onchocerciasis^8,51^ and for LF^25,30–32^, suggesting that this inverse relationship with the vector biting rate may represent a general feature of filarial infections. It also supports our proposal for the critical importance of including vector control in MDA programs against these diseases^30^, because as vector populations are reduced, infection breakpoints will increase commensurately in value until they are maximal at the TBR threshold applicable in a site^54^. The numerical values of the estimated site-specific mf breakpoints did not also differ statistically between the site-specific ABR versus focus ABR models, further supporting the central finding of this study, *viz*. that the latter models are able to satisfactorily capture the dynamics of infection in the investigated transmission zones of each of the present foci.

Although we found that values of a majority of the model parameters (up to 19 out of 22) were successfully updated by the site-specific data, highlighting the important role that data-model assimilation methods can play in improving scientific knowledge of complex parasite transmission systems, a significant finding is that only five parameters - all related to exposure - were sensitive to the type (site-specific or focus-level) of ABR used in simulating the present models. Despite this, both the site-specific and their corresponding focus-based models were able to adequately reproduce the age infection profiles in each study site, again emphasizing that many different models may be behaviourally able to fit the data observed for a complex system to an acceptable level, with the behavioural parameter values dispersed widely through the parameter space. While this equifinality reveals that many combinations of initial conditions, parameter sets, and model behaviours are consistent with observations, especially when measurements are few^14,20^ the consequences of such equifinality is clear, *viz*. it will induce uncertainty in inference as well as prediction, which in many cases will be difficult to further reduce imposing a limit to the predictability and thus use of any complex model^16,18,19^. Note here also that a related outcome of such complexity is that the more complex a model (multi-components and parameters, many functional responses and feedback loops) the harder it will be to refute it^23^, indicating that confirming observations may not demonstrate the veracity of a model but merely supports its probability conditional on errors in model structure, calibration of parameters or other auxiliary conditions, and the period of data used in the evaluation.

By contrast, an intriguing finding is that local *S. neavei* – borne oncercherciasis transmission adaptation appears to be dependent only on a few biological parameters (Fig. 4). Our results show that primarily these were parameters capturing variations in host immunity, exposure, infection aggregation and excess vector mortality both between sites (Fig. 4A) and foci (Fig. 4B). As we pointed out previously^25^, such dependence on only a few “stiff” parameters for adapting to local transmission conditions may allow a complex, multiparameter, parasitic system to use the majority of its defining parameters to counter changes in the local environment, including to perturbations induced by interventions, potentially making onchocerciasis transmission highly resilient to such shocks. One the one hand, this result underlines the likely difficulty of disrupting *S. neavei* –borne onchocerciasis transmission easily; however, the dependence of such “stiff” parameters, particularly those related to immunity and exposure, on variations in site or focus-level worm prevalence/density or ABR (Table 3, Fig. 4B) on the other hand means that inducing changes in these locally adapted parameters, eg. by reducing ABR using vector control or increasing immunity either through nutritional means or vaccination, may not only facilitate the disruption of the observed between-site or focus heterogeneity in transmission but also make the local system commensurately susceptible or fragile to perturbations or shocks that take the system far outside its (these) normal constraining conditions (see below)^25^.

Our results from modelling the impact of currently used or proposed MDA-based interventions on the comparative timelines to extinction given spatial variation in mf breakpoints and parasite transmission dynamics between sites and foci has provided further insights into the policy implications of the outcomes of these transmission complexities. First, we show that model uncertainty and equifinality will result in distributions of extinction timelines in a site (Table 5), which although varying significantly between the focus and site-specific ABR models were not large enough to be practically meaningful. This suggests that either model may contain sufficiently behavioural models, and thus both are useful for providing adequate predictions of the impact of applied interventions. This result also implies that the strategy of monitoring *S. neavei* ABR at the focus rather than at the sentinel village-level as carried out by the UOEP is sufficient for developing models for carrying out simulations of the outcomes of interventions, obviating the need (and expense) for conducting vector monitoring at finer spatial locations. The second insight gained from our intervention modelling study relates to the finding that the predicted durations of control required to eliminate *S. neavei-*transmitted onchocerciasis did not vary appreciably between sites within each focus (Table 5). They also did not vary markedly between foci that exhibited the occurrence of similar pre-control prevalences in sentinel monitoring sites, such as in the case of the Itwara and Kashaoya Kitomi foci investigated here, with predicted intervention durations showing differential reductions for MDA-only strategies in the case of Bwindi. This outcome signifies that if transmission-comparable strata occurs at the higher focus level, then it may be possible to use simulations from a selection of sentinel sites from such strata to fully characterize endemically-comparable regions within and between foci. Our results specifically show that in such cases even a selection and monitoring/modelling of 5–6 sites from strata along river systems where the highest transmission intensity may be expected is sufficient to capture the worst-case scenario expected with regard to timelines to *S. neavei-*onchocerciasis extinction both within a given focus as well as between foci. This finding again supports the policy of using information from a few comparable sentinel sites with the highest baseline prevalences along riverine systems that support black fly populations for subsequent intervention monitoring and for making decisions regarding when area-wide elimination of *S. neavei* – transmitted onchocerciasis may have occurred in endemic foci in Uganda.

Despite the above large-scale spatial effects, it is clear that the durations of control required to achive *S. neavei-*onchocerciasis elimination were greatly influenced by the type of MDA-based intervention scenario carried out in a site (Table 5). We show that overall the least effective scenario is low coverage annual MDA without VC resulting in requiring variously between 15–31 years to achieve transmission interruption in these sites even at 80% MDA coverage (Table 5), while the most effective strategy is high coverage biannual MDA with VC needing only between 3–7 years of control (Table 5). The Uganda Onchocerciasis program is beginning to consider switching to the use of biannual MDA currently^55^, and the results here show that this change may bring about major savings in the durations of interventions required for achieving onchocerciasis elimination in the country, with the model predictions showing that up to some 7–17 years could be saved in the present sites with this approach compared to application of annual MDA alone (Table 5). Although the intervention forecasts made here require to be assessed with follow-up data in the present sites for evaluating their plausibility, it is critical to note that the projections depicted in Table 5 lie within the 15–19 years found empirically to be needed for reducing mf prevalence to near elimination levels in the field using annual MDA^7,56–59^. Although not definitive, this correspondence of our predictions with observations provide confidence that the present locality-specific models are capable of yielding empirically realistic results not just for quantifying timelines to *S. neavei-*onchocerciasis elimination in a site but also for comparing the dynamics of extinction by site and by intervention option.

There are two outcomes that require discussion regarding the present predictions pertinent to the inclusion of VC into anti-*Onchocerca* MDA programs. The first is that dosing rivers with Abate in conjunction with MDA can significantly bring down the years required to accomplish the elimination of *S. neavei* –transmitted onchocerciasis. This can reduce the duration of interventions by over half those required by using the corresponding annual or biannual MDA alone, eg. by reducing the number of years of interventions required to 7–12 years by inclusion of this form of vector control compared to 15–31 years for annual MDA alone and just 3–6 years compared to 8–14 years in the case using biannual MDA alone (Table 5). These represent dramatic effects, and we indicate that present onchocerciasis programs, particularly in *S. neavei* areas, should now begin to urgently consider including Abate-based (or another appropriate vector intervention option, such as vegetation clearance^60^ or use of lethal traps^61^) control in ongoing MDA programs if the prospects of successfully meeting the goal of onchocerciasis elimination over the immediate future are to be achieved in these settings (see also^62^). The second major effect of including vector control into MDA programs is highlighted by the ability of this joint strategy to reduce the variabity in the timelines to extinction predicted by the present models (Fig. 6 and Table 5). The results indicate that both within- and, interestingly, between-site variability in such predictions can be reduced compared to when using MDA alone strategies, suggesting that including VC into MDA programs may not only offer a more effective option for eliminating onchocerciasis but also that it may overcome the effects of spatial heterogeneity to the extent that even the impact of between-foci heterogeneity may be reduced with predictions of intervention durations converging similarly across all three foci (Table 5). As we previously noted, this remarkable outcome of including vector control into MDA programs is brought about not just by a reduction in the robustness of parasite transmission but also by the tipping of transmission dynamics into a more narrowed and more fragile region^25,30,49^, leading to convergence in responses to perturbations irrespective of initial site-differences in transmission regimes.

The traditional approach to dealing with spatial complexity in modelling and managing ecological phenomena has been to consider the need for deriving models for the scale at which the major spatial effects occur and in addressing the relevant spatially adaptive options for achieving desired outcomes over a geographic domain of interest^13,21,22^. Here, our contribution is to demonstrate that in parasitic systems effectively addressing the impact of spatial complexity on modelling infection dynamics and control over a spatial domain will also crucially depend on the scale at which the heterogeneous ecology of transmission occurs. Thus, for *S. neavei* – transmitted onchocerciasis, given that maximal transmission occurs along tracts and watersheds surrounding the major riverine habitats of black flies, discovery of models even from a few survey sites for this discrete homogeneous transmission tracts will allow characterization of the dominant representative transmission dynamics for such strata. That is, for this disease there is no need to go below such strata to finer cell-by-cell or village levels to characterize the transmission dynamics in a spatial setting. This can enhance modelling efficiency and predictive power given that models need to be built only at the higher aggregate level either by lumping or grouping together parameters from a few sampling sites within each zone or via regularization^63^, in which global functional relationships describing transfer functions that link model parameters with environmental or lanscape characteristics are estimated and used. The implications for onchocerciasis elimination management of the occurrence of spatial effects at higher hierarchical scales, on the other hand, are more straightforward as it simply implies that given the characteristics of the intervention in question (see above) management may need to be adaptive only at the foci level. While this conclusion may argue for enacting an heterogeneity-based adaptive management strategy, eg. tailoring durations of annual MDA in particular by baseline prevalence (or ABR intensity) at the focus level (Table 5), as noted above, an alternative non-adaptive but still heterogeneity-underpinned approach is to apply strategies, such as *S. neavei* control using Abate applications, that could counter such spatial variability so that a stategy that works equally well everywhere can be deployed. Such a heterogeneity-based strategy may be simpler to implement and manage than adapting control by foci, and we have shown previously how including vector control in MDA programs for vector-borne macroparasitic diseases can also offer a resilient strategy that can sustain the eliminated state by raising the thresholds for re-emergence of infection^54,64^.

This study shows that charaterizing and modelling the impact of spatial heterogeneity and complexity will be critical to gaining a better understanding of the transmission dynamics and management of the area-wide elimination of complex vector-borne macroprasitic diseases, such as *S. neavei*-transmitted onchocerciasis. We also show that modelling frameworks that couple spatial datasets with data-model assimilation methods will be required if the appropriate scale-dependent local models are to be discovered and used to faciliate the making of predictions of the impact of implemented or proposed interventions that take a fuller account of spatial complexity. Our results also indicate that deriving and testing the effectiveness of heterogeneity-based approaches, as opposed to the normal homogeneity-focused command-and-control management model^40,65^, as an alternative paradigm for eliminating *S. neavei-*transmitted onchocerciasis, and indeed the other parasitic diseases chosen for global intervention, are now in order if we are to effectively achieve the goal of accomplishing area-wide control or elimination of these invariably spatially complex diseases successfully. We suggest that the development of data-driven modelling frameworks that pay careful attention to addressing the impacts of spatial patterns and factors that underlie the observed transmission dynamics of parasitic infections across a spatial domain will be critical to supporting the successful execution of the above task.

## Methods

### Data

The data used in this study were collected from sentinel field sites in three onchocerciasis endemic foci in western Uganda, *viz*. Itwara, Kashoya Kitomi, and Bwindi, as part of the monitoring and evaluation activities associated with the Ugandan Onchocerciasis Elimination Program (UOEP). While onchocercal transmission foci can extend to a range of distances from the fly source rivers, the sentinel villages for monitoring the impacts of interventions in Uganda are normally selected among those closest to these rivers to ensure that communities with the highest prevalence are longitudinally monitored to reflect impact in likely worse-case settings within a focus^45^. Note that while this design is efficient for monitoring the effects of an intervention in a focus, it will also reduce the potential extent of between-site infection/transmission heterogeneity among the chosen sentinel sites investigated in this work as compared to villages chosen randomly throughout the spatial extent of each focus.

Altogether, data assembled from sixteen such sites were selected for use in the present analysis based on the variable availability of baseline and follow-up microfilariae (mf) prevalence data, focus-level black fly annual biting rate (ABR) data at baseline, and details of interventions applied in each focus (Table 1 and Supplementary Table S7). Of these sites, eleven were from the hyperendemic Itwara and Kashoya Kitomi foci. The baseline mf prevalence in these villages ranged from 76.3% to 92.1%, and the black fly species responsible for transmission of *Onchocerca volvulus* in both foci is *Simulium neavei*. Note, however, that follow-up monitoring data from 2004 were available for only six of the sites (Table 1).

Initially, control efforts in these foci and across Uganda relied solely on annual rounds of community directed treatment with ivermectin (CDTi), but efforts were then scaled up to include vector control^66^. Successful elimination of the vector from several foci in Uganda using ground applications of temephos (Abate) eventually also shifted the focus of the program to transmission elimination rather than just control^66^. Thus, control activities in the Itwara focus specifically included 19 rounds of annual mass drug administration (MDA) with ivermectin from 1991–2009, with inclusion of vector control by ground larviciding at intermittent periods beginning from mid-1995 – June 2003^56,67^. In the Kashoya Kitomi focus, annual MDA with ivermectin began in 1991 and continued through 2006. In 2007, control efforts in this focus transitioned to biannual MDA, which continued through 2013 (Table 1). Vector control by ground larviciding was implemented here in July 2007 – October 2010^68^. Site-specific MDA coverage observed for the six villages from these foci that have follow-up data are given in Supplementary Table S7.

Impacts of vector control were investigated by UOEP by measuring changes in the aggregate values of ABR in each focus. Typically, biting data from human landing catches (HLCs) carried out in 14 catching sites along the relevant river system in a focus during the 4-5 months pertaining to the major transmission season (at frequencies of 2 days per week) were used to make these focus-level ABR calculations using the calculation methods described in^69^.

In addition, five villages from Bwindi, most of which are hypo- and mesoendemic, were also included in this study to consider a more heterogeneous sample of sites in the analyses (Table 1). For these sites, baseline mf infection data are aggregated at the community level and there was no biting rate information available (Table 1), requiring us to use model fits to the mf prevalence data to estimate the corresponding ABR information. Transmission here is also mediated by *S. neavei*, and both MDA and vector control interventions were carried out in this focus (Supplementary Table S7).

### The Onchocerciasis transmission model

The onchocerciasis transmission model used here is an immigration-death ecological model describing population level parasite transmission dynamics in both the human and black fly hosts. The general structure of the model follows that incorporated in previous deterministic population models of onchocerciasis transmission and control dynamics^47,70–75^, although here we develop and include functions describing the population-averaged mf uptake and larval development in the vector as well as the operation of different forms of host immunity in populations, while model implementation is carried out within a Bayesian Monte Carlo data-model assimilation framework that facilitates the simultaneous incorporation of information regarding local transmission parameters from data and assessment of the impact of uncertainty in the values of these parameters on model outputs^31,38,49,50,76–78^.

Technically, the present model is based on a hybrid coupled partial and ordinary differential equation system, where the population-level age-structured pre-patent (*P*) and patent (*W*) worm burdens, microfilarial counts (*M*) (mf/mg of skin), and acquired immunity level (*I*) in the human host are dynamically modelled by a set of partial differential equations over time (*t*) and age (*a*), whereas the dynamics of infective stage L3 larvae (*L*) in vector hosts (simuliid fly populations) are modelled by an ordinary differential equation over time. To reflect the significantly faster time-scale of the infection dynamics in the vector hosts, we, however, make the simplifying assumption that the density of infective stage larvae in the vector population reaches a dynamic equilibrium rapidly (L*)^70,75^. The governing equations of this model are:

$$\begin{array}{c}\begin{array}{c}\frac{\partial P}{\partial t}+\frac{\partial P}{\partial a}=\Phi {L}^{\ast }{F}_{1}(I(a,t)){F}_{2}({W}_{T}(a,t))-{\mu }_{w}P(a,t)-\Phi {L}^{\ast }{F}_{1}(I(a,t-\tau )){F}_{2}({W}_{T}(a,t-\tau ))\zeta \end{array}\\ \begin{array}{c}\frac{\partial W}{\partial t}+\frac{\partial W}{\partial a}=\Phi {L}^{\ast }{F}_{1}(I(a,t-\tau )){F}_{2}({W}_{T}(a,t-\tau ))\zeta -{\mu }_{w}W(a,t)\end{array}\\ \begin{array}{c}\frac{\partial M}{\partial t}+\frac{\partial M}{\partial a}={F}_{3}({W}_{T}(a,t))-\gamma M(a,t)\end{array}\\ \begin{array}{c}\frac{\partial I}{\partial t}+\frac{\partial I}{\partial a}={W}_{T}(a,t)-\delta I(a,t)\end{array}\\ \begin{array}{c}{L}^{\ast }={F}_{4}({W}_{T}(a,t))\end{array}\end{array}$$

Apart from multiple interacting components, complexity in the model is also governed by the operation of several density-dependent functional forms (denoted by the terms *F*_*x*_) that moderate the transitions or development rates of different stages in the parasite’s life cycle. These regulatory processes underpin the operations of two forms of host immunity, namely acquired immunity to incoming larvae (*F*_1_[*I*(*a*,*t*)]) and host immunosuppression by existing infection (*F*_2_[*W*_*T*_(*a*,*t*)]), mf production by patent worms (*F*_3_[*W*(*a*,*t*)]), and the development of infective stage larvae in the vector (*F*_4_[*M*(*a*,*t*)]). The larval death rate (*σ*_*L*_) and excess vector mortality (*σ*_*e*_) due to infection^47^ are also both considered in *F*_4_. Note that some functions are dependent on the total worm load where *W*_*T*_* = W(a,t) + P(a,t)*, while others depend on larval states (L*) and host immunity (*I*). To capture the effects of worm patency, we consider that at any given time *t*, human individuals of age less than or equal to pre-patency period, $\tau ,$ will have no adult worms or microfilariae, ie. *W*(*a*,*t*) = *M*(*a*,*t*) = 0 for *a *≤* τ*, and the rate at which pre-patent worms survive to become adult worms in these individuals at *a* > *τ* is given by $\zeta ={e}^{-{\mu }_{W}\tau }$. The establishment rate of larvae in the human host, represented as Φ, is moderated by effects of acquired immunity (as modelled by function, *F*_1_[*I*(*a*,*t*)]) and/or immune tolerance (modelled by function, *F*_2_[*W*_*T*_(*a*,*t*)])^73,74^. Given that previous modelling studies have underscored the potential for the involvement of either or both acquired immunity and immunosuppression in filarial infections^47,73,75^, we include both these types of immunity in our model; however, we do not make any *a priori* assumptions concerning their occurrence in a particular study site but instead use the mf prevalence data to determine the operation of each or both in a site (see discussion of data-assimilation using the Bayesian Melding framework below). The mathematical representation of adult *Onchocerca volvulus* worm mortality has recently been a topic of discussion^7^; here, we use a constant mortality rate following the example of Basañez and Boussinesq^47^ and Filipe *et al*.^75^. The vector biting rate (*λ*, a parameter in the function Φ) in this model considers the number of bites per black fly to be equal to the human blood index (*H*_*b*_) divided by the gonotrophic cycle (*g*). All model parameters and their descriptions are given in Supplementary Table S8. The respective density-dependent functions expected to modify or regulate transmission are given in Supplementary Table S9.

### Species-specific microfilariae uptake and L3 larval development

There exists a non-linear relationship between the uptake of mf by the black fly and the resulting L3-stage larval load^79^. This relationship will be dependent upon whether or not the relevant black fly species has developed cibarial armature, which we capture in the establishment rate term *f[M(a,t)]*. Following facilitation and limitation model formulations we developed previously^32^, the population-level mf uptake and larval establishment rate function for black flies with cibarial armature can be written as:

$$f[M(a,t)]=[\frac{2}{{[1+\frac{M(a,t)}{k}(1-\exp [-\frac{r}{\kappa }])]}^{k}}-\frac{1}{{[1+\frac{M(a,t)}{k}(1-\exp [-\frac{2r}{\kappa }])]}^{k}}]$$

whereas for black flies without developed cibarial armature we used the form:

$$f[M(a,t)]={(1+\frac{M(a,t)}{k}(1-\exp [-\frac{r}{\kappa }]))}^{-k}$$

In the above, $k={k}_{0}+{k}_{Lin}M$ is the shape parameter of the negative binomial distribution on the mf uptake whereas *r* and *κ* are respectively the rate of initial increase and the maximum level of L3 larvae. Parameter priors for *r* and *κ* were derived by fitting these functions to human and vector infection data from^80,81^. In this work, the relevant vector species, *S. neavei*, does not have well-developed cibarial armature, so we use the latter of the two formulations (Supplementary Table S9).

### Complex model discovery using data assimilation

Model-data fusion (also referred to as ‘data assimilation’ or ‘inverse modelling’) is a method whereby observations are used to optimize a model and quantify its uncertainty for a given setting^34–36^. Recent work has shown how such approaches via their ability to combine models with local data in a statistically rigorous manner can incorporate the effects of local initial conditions and input drivers to constrain model parameters and quantify model error^30,82^, thereby improving the discovery of behaviourally acceptable complex dynamical models applicable to a given setting^20^. Here, we build on one such strategy that facilitates the integration of field observations on onchocerciasis baseline mf prevalence and ABR in sentinel communities with simulation model outputs to estimate the values (and uncertainty) in the model parameters applicable for reproducing local parasite transmission dynamics. We then apply the locally calibrated model to undertake: (1) quantification of the infection endpoints or elimination thresholds applicable in each setting; and (2) based on model predicted mf prevalence trajectories, examine the effectiveness of various MDA-based intervention programs for breaking locality-specific parasite transmission. We used the model calibration and simulation framework founded on the Bayesian Melding (BM) algorithm to solve this local model discovery and prediction problem^38,50,76,77,83,84^, as follows.

The BM approach may be succinctly described as a procedure whereby two *priors* on a model output are compared and “melded” together in order to obtain the posterior parameter space in which the model may reliably explain the underlying natural system dynamics^84,85^. One of the priors on model output is the set of observed data; for example, in our case the survey data on onchocerciasis baseline mf prevalence collected from each endemic community before the start of a mass drug treatment program. The other output prior is the model-predicted values of the state variables, such as *W* or *M*, generated by values of initially-set parameters. Thus, the BM procedure is a method for reconciling several sources of prior information (on both input parameters and on model outcomes relative to data) to constrain the acceptable solution space of the model parameters. In the present implementation, we initially assigned uniform prior distributions for each of the model parameters to reflect our initial incomplete knowledge regarding their local values. The black fly biting rate servers as the primary driving variable, and is normally fixed to the values of the monthly biting rate (MBR) measured at baseline, although we can use the best-fitting models to age-infection data to quantify this variable in cases, such as for the sites in Bwindi, where these data are missing (see below and^4^). For assessing the reasonableness of model outputs to data, a binomial likelihood function was constructed to capture the distribution of the observed age-mf prevalence data, as follows:

$$L(\theta )=\mathop{\Pi }\limits_{g}\frac{{S}_{g}!}{{S}_{g}!({S}_{g}-{M}_{g})!}{{P}_{g}}^{{M}_{g}}{(1-{P}_{g})}^{({S}_{g}-{M}_{g})},$$

where $\theta$ represents a set of the model parameters, termed as parameter vector; ${M}_{g}$ denotes the total mf positive samples out of the total ${S}_{g}$ blood samples collected from people in the ${g}^{th}$ age-group, and the term ${P}_{g}$ is the modelled mf prevalence in the same age-group.

The BM method is essentially an iterative approach to inference, whereby our beliefs regarding a suitable model are updated based on the likelihood of new data, generating a posterior distribution and predictive interval for the observed data. The procedure we used is summarized as follows. There are 27 parameters in the model (Supplementary Table S8), which together form an initial prior parameter vector, ${\theta }_{i}$. Let the i^*th*^ element of a single parameter vector ${\theta }_{i}$ be defined by ${\theta }_{ii}$, ie, ${\theta }_{ii} \sim U[{\theta }_{ii}^{min},\,{\theta }_{ii}^{max}]$, where ${\theta }_{ii}^{min}$ and ${\theta }_{ii}^{max}$ are, respectively, the minimum and maximum prior values of that element parameter ${\theta }_{ii}$. With each of these parameter vectors, ${\theta }_{i=1,2,\ldots ,N=200,000}\in \Theta$, the model was simulated, and the corresponding distribution of model parameters $\pi (\theta )$ obtained. We then used the sampling-importance-resampling (SIR) algorithm to resample, with replacement, from the above set of *N* parameter vectors with the probability of acceptance of each resample ${\theta }_{j=1,2,\ldots ,l}$ probable to its relative weight ${\Omega }_{j}$, which is proportional to the corresponding likelihood $L({\phi }_{j})$ for the data, *i.e*. ${\Omega }_{j}=L({\phi }_{j})/{\sum }^{}L({\phi }_{i})$. A typical value of *l* for the results presented in this paper was around 500, and these posterior parameter sets are then used to generate distributions of variables of interest from the model (*e.g*. locality-specific age-prevalence curves, worm or mf breakpoints, and post-treatment infection trajectories), including measures of their uncertainties^31,49^.

### Calculation of worm breakpoints

Briefly, we applied a numerical stability analysis approach based on varying initial values of L^*^ (see details of the procedure provided in^32,49^), to each of the SIR-selected model vectors in order to calculate the distribution of mf prevalence breakpoints and threshold biting rates (TBR) expected in each study community. There are two essential steps in this approach. In the first step, we progressively decrease $\frac{V}{H}$ (the average number of black flies per human) from its original value to a threshold value below which the model always converges to zero mf prevalence, regardless of the values of the endemic infective larval density L^*^. The product of $\lambda$ and this newly found $\frac{V}{H}$ value is termed as the threshold biting rate (TBR). Once the threshold biting rate is discovered, the model at TBR can settle to either a zero (trivial attractor) or non-zero mf prevalence depending on the starting value of L^*^. Therefore, in the next step, while keeping all the model parameters unchanged, including the new $\frac{V}{H}$, and by starting with a very low value of L* and progressively increasing it in very small step-sizes we estimate the minimum L* below which the model predicts zero mf prevalence and above which the system progresses to a positive endemic infection state. The corresponding mf prevalence at this new L^*^ value is termed as the worm/mf breakpoint^32^. Note that a similar procedure can also be used to estimate the mf prevalence breakpoints at the undisturbed ABR values in a site. In this case, the estimated mf breakpoint prevalences will always be lower than the corresponding maximal values at TBR^25,30^. We thus use the mf breakpoint prevalences identified at ABR for simulating parasite elimination when MDA alone is used, while we employ the breakpoint values obtained at TBR when predicting transmission interruption as a result of including vector control with MDA^25,30^. The distribution of mf breakpoints thus quantified at either ABR or TBR values can finally be described by an empirical inverse cumulative density function^25,30^. We used this density function along with exceedance calculations to quantify the values of mf breakpoint prevalence thresholds reflecting a 95% elimination probability for use as the infection elimination target in this work. We used both the observed focus-level ABRs well as the estimated site-specific ABRs as required (see below) to estimate the site-specific worm/mf breakpoint and TBR values used in this analysis.

### Estimating site-specific ABRs

Previous studies have shown that the key environmental driver that governs pattern-process relationships in the case of vector-borne macroparasitic infections, and hence that primarily constrains a model’s parameter space so that the effects of local transmission conditions are captured, is the vector biting rate obtaining in a community^4,7,25,30^. However, in this study, the baseline data for each study village included information on this driving variable only at the higher aggregate focus level as described above. Although the use of such aggregated ABR to obtain the best-fit models within each sentinel village in a focus may offer a second-best option in the case of lack of information on vector abundance at the local level, it is apparent that this approach could introduce significant bias if the sentinel villages differed markedly in their local ABR values. We examined this possibility by using the models fitted to the mf prevalence data in each site in order to 1) estimate the corresponding site-specific ABRs, and 2) following this, undertaking a comparison of the values obtained against each measured focus-level ABR. To perform this exercise, each of the randomly sampled parameter vectors was used to obtain plausible ABR values by running the model under a standard root finding algorithm for which the model-generated overall mf prevalence matched the observed baseline community mf prevalence within a tolerance limit of 0.001 or lower. This root-finding algorithm was implemented in Matlab using its built-in *fzero* function^4^. The same procedure was also used to estimate the missing ABR data for the Bwindi sites.

### Modeling the effects of mass drug administration and vector control

The impact of MDA was modelled by assuming that anti-filarial treatment with the currently used ivermectin drug regimens acts by killing certain fractions of the populations of adult worms and mf instantly after drug administration, as well as reducing the fertility of surviving female adult worms^86–88^. We denote the fractions killed as *ω* for adult worms, and *ε* for mf. The population sizes of worms and microfilariae are calculated after drug treatment by modifying the populations of each parasite stage obtained immediately prior to the treatment by:

$$\begin{array}{c}P(a,t+dt)=(1-\omega C)P(a,t)\\ W(a,t+dt)=(1-\omega C)W(a,t)\\ M(a,t+dt)=(1-\varepsilon C)M(a,t)\end{array}\}{\rm{at}}\,{\rm{time}}\,t={T}_{MDAi}$$

In the above, $dt$ represents a short time-period since the time-point ${T}_{MD{A}_{i}}$ when the *i*^*th*^ MDA was administered. During this short time-interval, a given proportion of pre-patent/adult worms and mf (as specified by values of the drug efficacy rates for these life stages, $\omega$ and $\varepsilon ,$ respectively) are instantaneously killed. The parameter *C* is the population-level drug coverage in this formulation (note that the population coverage is applied to individuals ≥ 5 years old as children < 5 years old are ineligible for ivermectin treatment). Apart from instantaneous killing of adult worms and mf, ivermectin is also thought to sterilize or suppress the production of mf by worms surviving each MDA^89^. Here we modeled this effect by introducing a new parameter (denoted by *δ*_*reduc*_) as follows:

$$\frac{\partial M}{\partial t}+\frac{\partial M}{\partial a}=(1-{\delta }_{reduc}C)s\alpha \phi [W(a,t),k]W(a,t)-\gamma M(a,t),\,{\rm{for}}\,{T}_{MDAi} < t\le {T}_{MDAi}+P$$

where $\alpha {\prime} =\alpha (1-{\delta }_{reduc}C)$ reflects the suppressed fecundity (over a period of *T*_*P*_ months since the *i*^*th*^ MDA) of adult female worms that survive the administration of ivermectin at each MDA.

We consider that integrating vector control (VC) using Abate with MDA will primarily add to the effects of MDA on worms by causing a reduction in the baseline black fly biting density over time. The decline in the black fly biting density ($\frac{V}{H}$) is modeled as an exponential decay process, *viz*. $\frac{V}{H}(1-\exp (\,-\,{A}_{0}t){C}_{VC})$ where ${A}_{0}$ is the expected decline in the biting density in the study areas undergoing Abate-based VC (Supplementary Fig. S3). The VC coverage parameter (*C*_*VC*_) takes the value of unity for the case when all black fly breeding habitats are treated with larvicides. The values of instant worm/mf killing and suppression of mf production by surviving worms due to ivermectin, and the vector biting decay rate estimated from field data portraying observed declines in fly biting after larvicide application (${A}_{0}$) are given in Supplementary Table S10.

The first MDA round is implemented as reducing the predicted baseline parasitic infection intensities (i.e. densities of worms and microfilariae) at the time of treatment. Infection dynamics are then tracked over time using a monthly time step, with the remaining MDAs implemented according to an annual and/or biannual treatment schedule. The impact of VC is modelled as reducing the biting rate starting from the first month of larvicide treatment. We simulated the effects of interventions under four hypothetical scenarios (annual and biannual MDAs without/with VC) with drug coverages ranging from 40% to 100%. Predictions arising from MDA provided at 80% coverage served to reflect the optimal scenario in these analyses. In addition, we also simulated the outcomes of the observed interventions carried out in the six sites where follow-up mf prevalence data were available. In this analysis, for years in which the site-specific MDA coverage was not available, we applied the observed coverage at the focus level, if available (2006–2008 in Itwara sites and 2006–2010 in the Kashoya Kitomi sites) (Supplementary Table S7). In the absence of any information, as was the case for 2011–2013 in Kashoya Kitomi, we assumed the coverage achieved in the previous year was maintained.

### Statistical tests for analysing data and model-estimated quantities

A pass/fail scoring approach was used to test for the goodness-of-fit of the SIR-selected models to the baseline mf age-prevalence data^90^. A model was considered behavioral if, for at least all but two age groups, the predicted prevalence for a given age group was within the 95% binomial confidence interval bounds for the observed data. The proportion of SIR model fits that fell with these bounds were scored to give an index of good-fit. Differences in the prior and posterior distributions of model parameters were tested by the 2-sample Kolmogorov-Smirnov (KS) test using the *kstest2* function in Matlab. Classification trees for identifying important variables underlying between site heterogeneities were built and analyzed using the R package *rpart*. The binomial generalized additive model for evaluating differences in baseline data between either sentinel villages or foci were carried out using the *mgcv* package in R. Kruskal-Wallis tests for difference in model-estimated quantities (*e.g*. mf breakpoints, timelines to elimination) between locations and/or models were carried out using the Matlab function *kruskalwallis*.

### Availability of materials and data

All data analysed during this study are included in this published article and its supplementary information files. Code for running the onchocerciasis model is available at https://github.com/EdwinMichaelLab/OnchoModel_Uganda.git.

## Supplementary information

Supporting Information.
