# Supplementary material for: Data-driven modelling and spatial complexity supports heterogeneity-based integrative management for eliminating Simulium neavei-transmitted river blindness
Source: Sci Rep. 2020 Mar 6;10:4235. doi: 10.1038/s41598-020-61194-w (PMC7060237; doi:10.1038/s41598-020-61194-w)
Supplement: Supplementary file 1 — Supporting Information. [file 41598_2020_61194_MOESM1_ESM.docx]

Supporting Information:

Data-driven predictive modelling, spatial complexity, and the management of *Simulium neavei*-transmitted river blindness elimination

Edwin Michael, Morgan E. Smith, Brajendra K. Singh, Moses N. Katabarwa, Edson Byamukama, Peace Habomugisha, Thomson Lakwo, Edridah Tukahebwa, Frank O. Richards

**Supplementary Table S1. Percentage of model predictions falling within the 95% binomial confidence intervals of the observed baseline mf prevalence data.**

| Sentinel village | Passing predictions (%) |
| --- | --- |
| Byeya | 74.4 |
| Igoma | 100 |
| Kajuma | 95.2 |
| Kakira | 99.4 |
| Kibangali | 98.6 |
| Mirambi | 99.6 |
| Buhanda | 100 |
| Ihunda | 100 |
| Kakasi | 100 |
| Kengeyo | 92.4 |
| Nsinde | 98 |

**Supplementary Table S2. Comparison of focus and site-specific ABR model performance through the relative RMSE metric.**

| Sentinel village | ReRMSE | |
| --- | --- | --- |
|  | Focus ABR models | Site-specific ABR models |
| Byeya | 1.0042 | 1.0042 |
| Igoma | 1.0018 | 1.0018 |
| Kajuma | 1.0132 | 1.0133 |
| Kakira | 1.0201 | 1.0200 |
| Kibangali | 1.0074 | 1.0075 |
| Mirambi | 1.0043 | 1.0043 |
| Buhanda | 1.0193 | 1.0192 |
| Ihunda | 1.0273 | 1.0273 |
| Kakasi | 1.0364 | 1.0362 |
| Kengeyo | 1.0117 | 1.0117 |
| Nsinde | 1.0046 | 1.0046 |

Smaller ReRMSE values indicate better model fit to data.

**Supplementary Table S3. Kolmogorov-Smirnov test results for differences in prior and posterior parameter distributions in each village given the site-specific ABR models.**

| Sentinel  village | Model parameter^3^ | | | | | | | | | | | | | | | | | | | | | |
| --- | --- | --- | --- | --- | --- | --- | --- | --- | --- | --- | --- | --- | --- | --- | --- | --- | --- | --- | --- | --- | --- | --- |
|  | H^b^ ^1^ | g | α | k_0_ | k_Lin_ | κ | r | σ | ψ_1_ | ψ_2_ | μ_w_ | γ | b | c | H_Lin_ | I_C_ | S_C_ | I_Cmin_ | σ_L_ | τ | δ | σ_e_ |
| Byeya | 1^2^ | 1 | 1 | 0 | 1 | 0 | 0 | 1 | 1 | 1 | 0 | 0 | 1 | 1 | 1 | 1 | 1 | 1 | 0 | 1 | 0 | 1 |
| Igoma | 1 | 1 | 1 | 0 | 1 | 0 | 0 | 1 | 1 | 1 | 1 | 1 | 1 | 1 | 1 | 1 | 1 | 1 | 0 | 0 | 0 | 1 |
| Kajuma | 1 | 1 | 1 | 0 | 1 | 0 | 0 | 1 | 1 | 1 | 1 | 1 | 1 | 1 | 1 | 1 | 1 | 1 | 0 | 1 | 0 | 1 |
| Kakira | 1 | 1 | 1 | 0 | 1 | 0 | 0 | 0 | 1 | 1 | 1 | 1 | 1 | 1 | 1 | 1 | 1 | 1 | 0 | 0 | 1 | 1 |
| Kibangali | 1 | 1 | 1 | 0 | 1 | 0 | 1 | 0 | 1 | 1 | 1 | 1 | 1 | 1 | 1 | 0 | 0 | 1 | 0 | 0 | 1 | 1 |
| Mirambi | 1 | 1 | 1 | 1 | 1 | 0 | 1 | 1 | 1 | 1 | 0 | 0 | 1 | 1 | 1 | 1 | 1 | 1 | 0 | 1 | 0 | 1 |
| Buhanda | 1 | 1 | 1 | 0 | 1 | 0 | 0 | 1 | 1 | 1 | 0 | 0 | 1 | 1 | 1 | 1 | 1 | 1 | 0 | 0 | 0 | 1 |
| Ihunda | 1 | 1 | 1 | 0 | 1 | 0 | 0 | 0 | 1 | 1 | 0 | 0 | 1 | 1 | 1 | 1 | 1 | 1 | 0 | 0 | 0 | 1 |
| Kakasi | 1 | 1 | 1 | 1 | 1 | 0 | 1 | 1 | 1 | 1 | 0 | 0 | 1 | 1 | 1 | 1 | 1 | 1 | 0 | 0 | 0 | 1 |
| Kengeyo | 1 | 1 | 1 | 0 | 1 | 0 | 0 | 0 | 1 | 1 | 0 | 1 | 1 | 1 | 1 | 1 | 1 | 1 | 0 | 0 | 0 | 1 |
| Nsinde | 1 | 1 | 1 | 0 | 1 | 0 | 0 | 1 | 1 | 1 | 1 | 1 | 1 | 1 | 1 | 1 | 1 | 1 | 0 | 0 | 0 | 1 |
| Muko | 1 | 1 | 1 | 1 | 1 | 0 | 0 | 1 | 1 | 1 | 0 | 0 | 1 | 0 | 1 | 1 | 1 | 1 | 0 | 0 | 0 | 1 |
| Kashaka | 1 | 1 | 1 | 0 | 1 | 0 | 0 | 0 | 1 | 1 | 0 | 0 | 1 | 1 | 1 | 1 | 1 | 1 | 0 | 1 | 0 | 1 |
| Mugombwa | 1 | 1 | 1 | 0 | 1 | 0 | 0 | 0 | 1 | 1 | 1 | 0 | 1 | 1 | 1 | 1 | 1 | 1 | 0 | 1 | 1 | 1 |
| Kikobero | 1 | 1 | 1 | 0 | 1 | 0 | 1 | 0 | 1 | 1 | 1 | 1 | 1 | 1 | 1 | 1 | 1 | 1 | 0 | 1 | 0 | 1 |
| Suma | 1 | 1 | 1 | 1 | 1 | 0 | 0 | 1 | 1 | 1 | 0 | 0 | 1 | 1 | 1 | 1 | 0 | 1 | 0 | 0 | 0 | 0 |

^1^Shaded parameters did not differ from their priors across a majority (>8) of the sentinel villages.

^2^Parameters whose posterior distributions statistically significantly differed from their prior distributions (*p*-value < 0.05) according to the focus ABR models are identified with a 1, while those posteriors which did not differ from their priors are identified with a 0.

^3^See Supplementary Table S8 for parameter definitions.

**
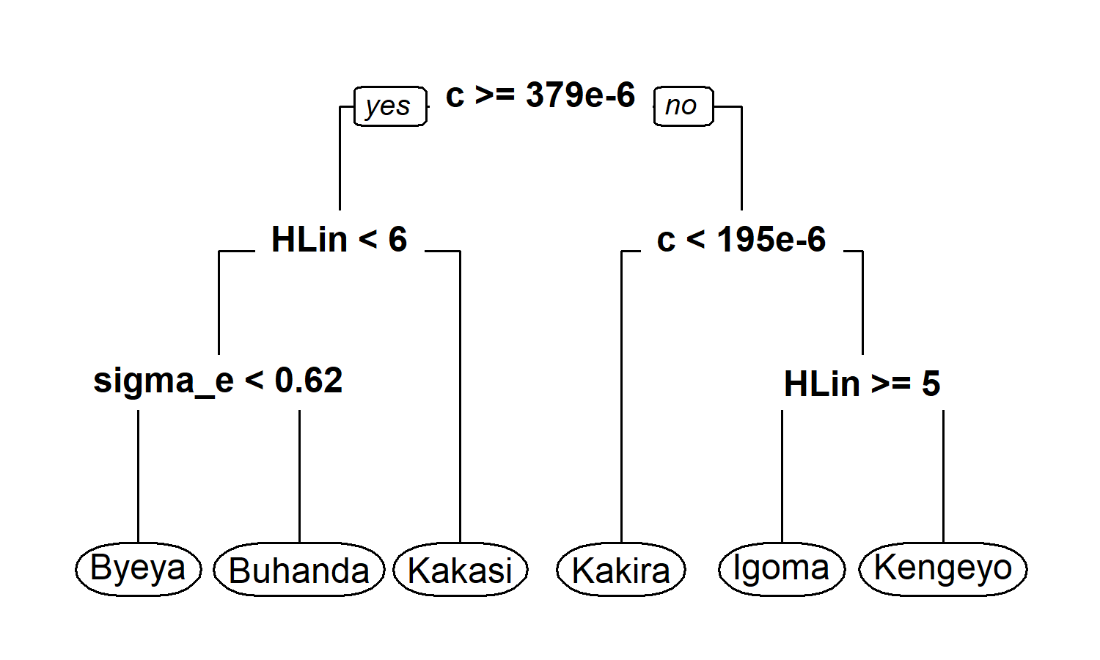
**

**Supplementary Figure S1. Classification tree highlighting the model parameters that were important for explaining differences between sentinel sites.** The best-fitting parameter sets for the site-specific ABR models were analyzed here. Villages were classified according to their fitted parameter values using the rpart package in R. The tree was pruned with the complexity parameter set to 0.005 and a tree depth of 3 is shown here. The top three variables by importance were c, H_Lin_, and σ_e_ (see Supplementary Table S8 for parameter definitions).

.

**
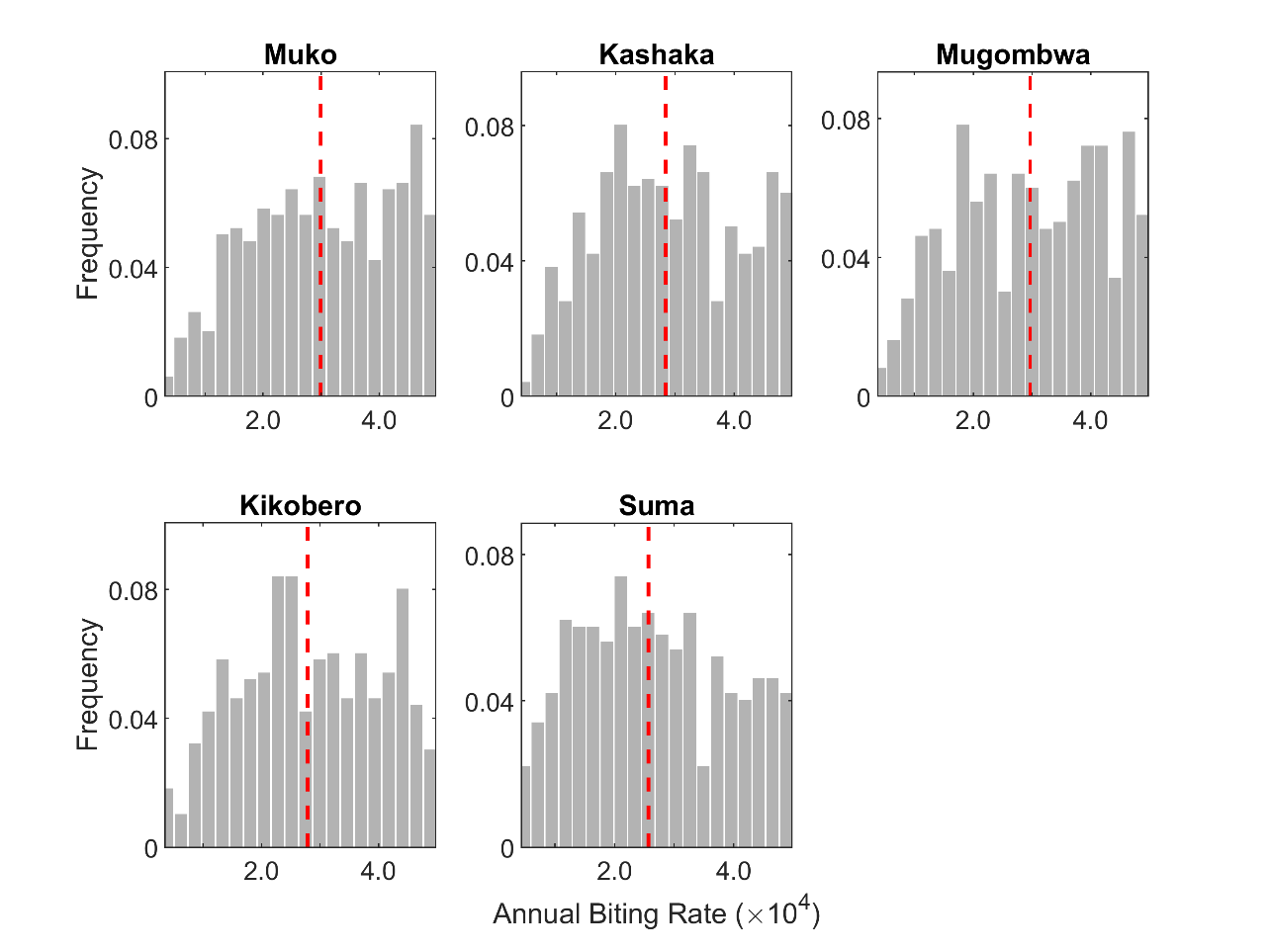
**

**Supplementary Figure S2. Model-estimated site-specific annual biting rates for sites in the Bwindi focus.** The histograms show the relative frequencies of the model-estimated annual biting rates for each village studied in the Bwindi focus. The red vertical dashed line indicates the median estimated ABR value. The estimated ABR distributions between sites are statistically significantly different as given by a Kruskal-Wallis test (*p*-value < 0.05).

**Supplementary Table S4. Kruskal-Wallis tests for comparing mf breakpoints estimated by the site-specific versus focus ABR models for each site.**

| Village | *p*-value | |
| --- | --- | --- |
|  | Mf breakpoint at ABR | Mf breakpoint at TBR |
| Byeya | **3.51 e-7** | 0.5875 |
| Igoma | **0.0421** | 0.1954 |
| Kajuma | **0.0070** | 0.9960 |
| Kakira | **0.0027** | **9.65 e-7** |
| Kibangali | 0.9870 | 0.6423 |
| Mirambi | 0.9308 | **2.34 e-5** |
| Buhanda | 0.2747 | 0.2855 |
| Ihunda | **0.0131** | **0.0228** |
| Kakasi | 0.0964 | **0.0189** |
| Kengeyo | **0.0379** | 0.4069 |
| Nsinde | 0.8271 | 0.1284 |

Bolded values highlight significant *p*-values (< 0.05).


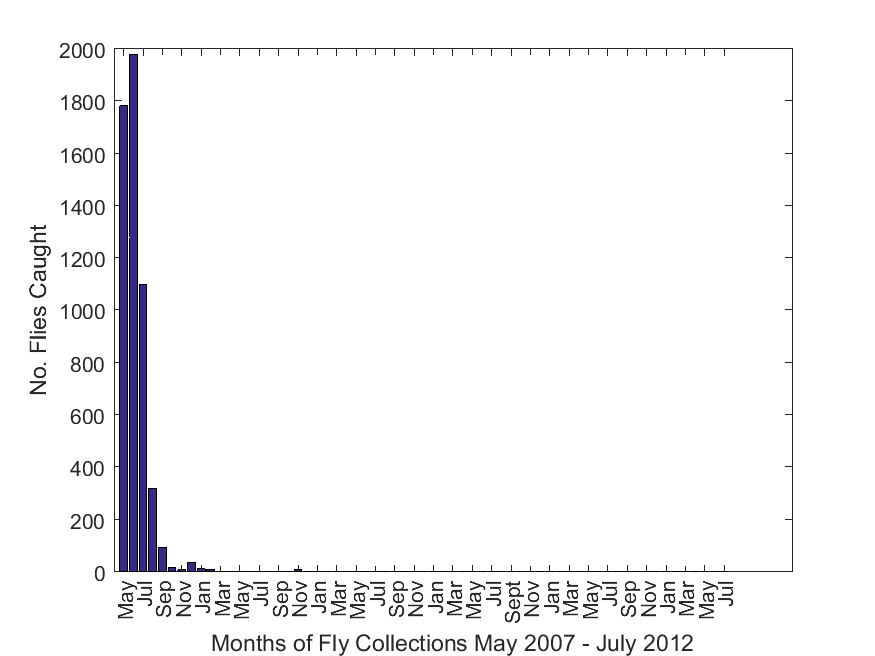


**Supplementary Figure S3. Fly catch data in Kashoya Kitomi.** An exponential decay function was fit to this data to determine the decay rate of fly biting activity as a result of the application of larvicide.

**Supplementary Table S5. Kruskal-Wallis test *p*-values for comparing timelines to reach 95% EP threshold between villages and transmission foci.**

| Intervention strategy | Focus ABR models | | Site-specific ABR models | |
| --- | --- | --- | --- | --- |
|  | Village | Focus | Village | Focus |
| Annual MDA | **< 2.2 e-16** | **< 2.2 e-16** | **< 2.2 e-16** | **< 2.2 e-16** |
| Annual MDA + VC | **< 2.2 e-16** | **3.40 e-4** | **< 2.2 e-16** | **< 2.2 e-16** |
| Biannual MDA | **< 2.2 e-16** | **< 2.2 e-16** | **< 2.2 e-16** | **< 2.2 e-16** |
| Biannual MDA + VC | **< 2.2 e-16** | **0.0046** | **< 2.2 e-16** | **< 2.2 e-16** |

Bolded values highlight significant *p*-values (< 0.05).

**Supplementary Table S6. Kruskal-Wallis tests for comparing timelines to reach 95% EP threshold given site-specific ABR versus focus ABR models for each site.**

| Village | p-value | | | |
| --- | --- | --- | --- | --- |
|  | Annual MDA | Annual MDA + VC | Biannual MDA | Biannual MDA + VC |
| Byeya | **9.72 e-6** | **< 2.2 e-16** | **8.20 e-13** | **< 2.2 e-16** |
| Igoma | **< 2.2 e-16** | 0.1944 | **< 2.2 e-16** | **0.0299** |
| Kajuma | **0.0430** | **< 2.2 e-16** | **1.78 e-9** | **< 2.2 e-16** |
| Kakira | **8.66 e-11** | **2.29 e-9** | **1.59 e-13** | **4.35 e-10** |
| Kibangali | **3.35 e-5** | **< 2.2 e-16** | **1.52 e-9** | **< 2.2 e-16** |
| Mirambi | **2.04 e-9** | **< 2.2 e-16** | **3.10 e-10** | **< 2.2 e-16** |
| Buhanda | **2.01 e-9** | **1.93 e-8** | **9.28 e-10** | **6.38 e-7** |
| Ihunda | **< 2.2 e-16** | **2.20 e-5** | **< 2.2 e-16** | **2.56 e-7** |
| Kakasi | **1.37 e-15** | **< 2.2 e-16** | **< 2.2 e-16** | **4.56 e-15** |
| Kengeyo | 0.3538 | **< 2.2 e-16** | 0.1784 | **< 2.2 e-16** |
| Nsinde | **< 2.2 e-16** | **< 2.2 e-16** | **< 2.2 e-16** | **< 2.2 e-16** |

Bolded values highlight significant *p*-values (< 0.05).

**Supplementary Table S7. Observed MDA coverage and use of supplemental vector control by year for six sentinel villages.**

| Sentinel Village | Intervention year | | | | | | | | | | | | | | | | | | | | | | |
| --- | --- | --- | --- | --- | --- | --- | --- | --- | --- | --- | --- | --- | --- | --- | --- | --- | --- | --- | --- | --- | --- | --- | --- |
|  | 1991 | 1992 | 1993 | 1994 | 1995 | 1996 | 1997 | 1998 | 1999 | 2000 | 2001 | 2002 | 2003 | 2004 | 2005 | 2006 | 2007^4^ | 2008 | 2009 | 2010 | 2011 | 2012 | 2013 |
| Byeya | 47^1^ | 58 | 60 | 68 | 70^2^ | 70 | 70 | 66 | 69 | 63 | 75 | 73 | 59 | 65 | 71 | 89^3^ | 87^3^ | 90^3^ | 80 | MDA stopped | | | |
| Kajuma | 35 | 45 | 57 | 61 | 64 | 63 | 67 | 58 | 61 | 63 | 55 | 69 | 70 | 71 | 78 | 89^3^ | 87^3^ | 90^3^ | 80 |  |  |  |  |
| Igoma | 51 | 61 | 58 | 60 | 71 | 69 | 65 | 66 | 65 | 57 | 68 | 72 | 68 | 70 | 73 | 89^3^ | 87^3^ | 90^3^ | 79 |  |  |  |  |
| Kengeyo | 53 | 55 | 59 | 64 | 68 | 74 | 79 | 80 | 81 | 80 | 76 | 75 | 84 | 76 | 72 | 76^3^ | 80^3^ | 80^3^ | 80^3^ | 80^3^ | - | - | - |
| Nsinde | 45 | 64 | 60 | 59 | 61 | 65 | 65 | 65 | 74 | 71 | 85 | 85 | 73 | 69 | 79 | 76^3^ | 80^3^ | 80^3^ | 80^3^ | 80^3^ | - | - | - |
| Ihunda | 56 | 68 | 61 | 60 | 69 | 67 | 75 | 79 | 80 | 86 | 61 | 61 | 88 | 85 | 86 | 76^3^ | 80^3^ | 80^3^ | 80^3^ | 80^3^ | - | - | - |

^1^MDA coverage represents the proportion of the total population which received treatment.

^2^Shaded cells indicate years in which vector control was implemented (1995-2003 in Itwara, and 2007-2010 in Kashoya Kitomi).

^3^MDA coverage from the focus level were applied when village level data was not available.

^4^The MDA frequency in Kashoya Kitomi switched from annual to biannual in 2007, and the MDA coverage given in from 2007 onwards is assumed to apply for both rounds of treatment.

**Supplementary Table S8. Description of model parameters.**

| **Parameter** | **Definition (units)** | **Prior parameter range** | **References** |
| --- | --- | --- | --- |
| *λ* | Number of bites per vector (per month) | $=\frac{H_{b}}{g}$ |  |
| *H_b_* | Human blood index | [0.3, 0.99] | [1-5] |
| *g* | Period of gonotrophic cycle (months) | [0.067, 0.13] | [3, 4, 6, 7] |
| *V/H* | Ratio of number of vectors to hosts | MBR^1^ / *λ* | data |
| *H_Lin_^2^* | Threshold value used in *h(a)* to adjust the age-dependent exposure rate (months) | [12, 240] | [8] |
| *A^2^* | Coefficient describing population age distribution in *π(a)* | data | [8] |
| *B^2^* | Coefficient describing population age distribution in *π(a)* | data | [8] |
| *ψ_1_* | Proportion of L3 leaving vector per bite | [0.12, 0.7] | [3-6, 9] |
| *ψ_2_* | Larval establishment rate^3^ | [0.02, 0.0854] | [3-6] |
| *c* | Strength of acquired immunity | [0.0001, 0.001] | [10, 11] |
| *I_Cmin_* | Baseline presence of immunosuppression | [0.0025, 1] | [10, 11] |
| *I_C_* | Strength of immunosuppression^4^ | [0.5, 5.5] | [10, 11] |
| *S_C_* | Slope of immunosuppression function^5^ (per worm/month) | [0.1, 0.75] | [10, 11] |
| *$\delta$* | Immunity waning rate (per month) | [0.00001, 0.0001] | [10, 11] |
| *μ_W_* | Worm mortality rate (per month) | [0.0083, 0.0104] | [3-6, 9] |
| *τ* | Pre-patency period (months) | [9, 26] | [6, 9, 12] |
| *k_0_* | Basic location parameter of negative binomial distribution used in *k* | [0.00036, 0.0044] | [10, 11] |
| *k_Lin_* | Linear rate of increase in *k* | [0.00000024, 0.282] | [10, 11] |
| *s* | Proportion of female worms | 0.5 | - |
| *α* | Production rate of microfilariae per worm (per month) | [0.25, 1.5] | [3-6, 9] |
| *γ* | Microfilariae mortality rate (per month) | [0.08, 0.12] | [3-6, 9] |
| *b* | Proportion of vectors which pick up infection when biting an infected host | [0.259, 0.481] | [3-6] |
| *κ* | Maximum level of L3 given Mf density | [1.16, 2.00] | [13, 14] |
| *r* | Gradient of Mf uptake^6^ | [0.01,0.0495] | [13, 14] |
| *σ* | Vector mortality rate (per month) | [1.5, 8.5] | [3-6] |
| *σ_e_* | Excess vector mortality due to mf infection (per month) | [0.75, 4.25] | [15] |
| ***σ_L_*** | Larval mortality rate | [0.33, 1.16] | [3-6] |

^1^Note MBR (monthly biting rate) serves as an input to initialize the model, measured as mosquito bites per person per month, the value of which may be obtained from entomological surveys conducted in study sites. In the absence of the observed MBR value, the model has been adapted to estimate it from the community-level Mf prevalence data.

^2^The parameters *A*, *B*, and *H_Lin_* are estimated from national human demographic data or from the age-prevalence data.

^3^The proportion of L3-stage larvae infecting human hosts that survive to develop into adult worms.

^4^The facilitated establishment rate of adult worms due to parasite-induced immunosuppression in a heavily infected human host.

^5^The initial rate of increase by which the strength of immunosuppression is achieved as *W* increases from 0 [11].

^6^The gradient of Mf uptake *r* is a measure of the initial increase in the infective L3 larvae uptake by vector as *M* increases from 0 [8].

**Supplementary Table S9. Description of model functions and functional forms.**

| **Function** | **Functional form** |
| --- | --- |
| $h(a)$: age-dependent exposure rate | $=\left\{\begin{matrix} \frac{a}{H_{Lin}}\text{ for }a<H_{Lin} \\ 1\text{ for }a\geq H_{Lin} \end{matrix}\right.$ |
| $k$: parasite aggregation | $=k_{0}+k_{Lin}\overline{M(a,t)}$ |
| $\zeta$: rate of pre-patent worm maturation | $=e^{-\mu_{W}\tau}$ |
| :worm mating probability | $=1-\left(1+\frac{W}{2k}\right)^{-(1+k)}$ |
| $\pi(a)$: population age distribution | $=Ae^{(-Ba)}$ |
| $f[M(a,t),k]$: Vector Mf uptake response |   for vectors with cibarial armature  for vectors without cibarial armature |
| $\Phi$: larval establishment rate | $=\lambda\frac{V}{H}h(a)\psi_{1}\psi_{2}$ |
| $F_{1}\left[I\left(a,t\right)\right]$ : human immunity to larval establishment | $=\frac{1}{1+cI(a,t)}$ |
| $F_{2}\left[W_{T}(a,t)\right]$ : human immunosuppression | $=\frac{I_{C\min}+I_{C}S_{C}W_{T}(a,t)}{1+S_{C}W_{T}(a,t)}$ |
| $F_{3}$: Mf production in the human host |  |
| $F_{4}$: L3 stage larval density in the vector | $=\frac{\lambda\kappa b\int\pi(a)(1-f[M(a,t),k])da}{\sigma_{L}+\sigma+\lambda\psi_{1}+\sigma_{e}\int\pi(a)h(a)M(a,t)}$ |

**Supplementary Table S10. Drug and vector control parameters**

| **Parameter** | **Definition** | **Prior parameter range** | **References** |
| --- | --- | --- | --- |
| *ω* | Worm killing efficacy (instantaneous) | [0.1, 0.3] | [16-23] |
| *ε* | Microfilariae killing efficacy (instantaneous) | [0.95, 0.99] | [16-19, 23] |
| *$\delta_{reduc}$* | Reduction in microfilariae production by surviving worms | [0.35, 0.75] | [16-19, 23] |
| *T_P_* | A time period during which the drug remains efficacious in reducing the fecundity of the surviving adult worms | 9 months | [17-19, 21, 22] |
| *C* | Percentage of human population administered ivermectin | data | data |
| *$A_{0}$* | The decay rate used in the exponential reduction in vector biting density | [0.2, 2.8] | data (Supp Fig S1) |
| *$C_{VC}$* | The population-level vector control coverage | 0: no VC  1: VC | data |

**References**

1. Garms R. Observations on filarial infections and parous rates of anthropophilic blackflies in Guatemala, with reference to the transmission of Onchocerca volvulus. Tropenmed Parasitol. 1975;26(2):169-82.

2. Ochoa A. Studies on the anthropophilic blackfly species in Guatemala, with special reference to the transmission of onchocerciasis in the southeastern endemic area. Jpn J Sanit Zool. 1982.

3. Basanez MG, Remme JHF, Alley ES, Bain O, Shelley AJ, Medley GF, et al. Density-Dependent Processes in the Transmission of Human Onchocerciasis - Relationship between the Numbers of Microfilariae Ingested and Successful Larval Development in the Simuliid Vector. Parasitology. 1995;110:409-27.

4. Basanez M-G, Boussinesq M. Population biology of human onchocerciasis. Philos Trans R Soc Lond B Biol Sci. 1999;354(1384):809-26.

5. Basanez MG, Collins RC, Porter CH, Little MP, Brandling-Bennett D. Transmission intensity and the patterns of Onchocerca volvulus infection in human communities. Am J Trop Med Hyg. 2002;67(6):669-79.

6. Filipe JA, Boussinesq M, Renz A, Collins RC, Vivas-Martinez S, Grillet ME, et al. Human infection patterns and heterogeneous exposure in river blindness. Proc Natl Acad Sci U S A. 2005;102(42):15265-70.

7. Basanez M-G, Razali K, Renz A, Kelly D. Density-dependent host choice by disease vectors: epidemiological implications of the ideal free distribution. Trans R Soc Trop Med Hyg. 2007;101(3):256-69.

8. Norman RA, Chan M-S, Srividya A, Pani SP, Ramaiah KD, Vanamail P, et al. EPIFIL: the development of an age-structured model for describing the transmission dynamics and control of lymphatic filariasis. Epidemiol Infect. 2000;124(03):529-41.

9. Duke BO. Observations and reflections on the immature stages of Onchocerca volvulus in the human host. Ann Trop Med Parasitol. 1991;85(1):103-10.

10. Duerr HP, Dietz K, Schulz-Key H, Büttner DW, Eichner M. Density-dependent parasite establishment suggests infection-associated immunosuppression as an important mechanism for parasite density regulation in onchocerciasis. Trans R Soc Trop Med Hyg. 2003;97(2):242-50.

11. Duerr H-P, Dietz K, Eichner M. Determinants of the eradicability of filarial infections: a conceptual approach. Trends Parasitol. 2005;21(2):88-96.

12. Duerr HP, Eichner M. Epidemiology and control of onchocerciasis: the threshold biting rate of savannah onchocerciasis in Africa. Int J Parasitol. 2010;40(6):641-50.

13. Grillet M-E, Villamizar NJ, Frontado HL, Cortez J, Escalona M, Botto C, et al. Vector competence of Simulium oyapockense and S. incrustatum for Onchocerca volvulus: Implications for ivermectin-based control in the Amazonian focus of human onchocerciasis, a multi-vector–host system. Acta Trop. 2008;107(2):80-9.

14. Soumbey-Alley E, Basanez M-G, Bissan Y, Boatin BA, Remme JHF, Nagelkerke NJD, et al. Uptake of Onchocerca volvulus (Nematoda: Onchocercidae) by Simulium (Diptera: Simuliidae) is not strongly dependent on the density of skin microfilariae in the human host. J Med Entomol. 2004;41(1):83-94.

15. Basáñez MG, Townson H, Williams JR, Frontado H, Villamizar NJ, Anderson RM. Density-dependent processes in the transmission of human onchocerciasis: relationship between microfilarial intake and mortality of the simuliid vector. Parasitology. 1996;113:331-55.

16. Awadzi K, Attah SK, Addy ET, Opoku NO, Quartey BT. The effects of high-dose ivermectin regimens on Onchocerca volvulus in onchocerciasis patients. Trans R Soc Trop Med Hyg. 1999;93(2):189-94.

17. Coffeng LE, Stolk WA, Hoerauf A, Habbema D, Bakker R, Hopkins AD, et al. Elimination of African Onchocerciasis: Modeling the Impact of Increasing the Frequency of Ivermectin Mass Treatment. PLoS One. 2014;9(12):e115886.

18. Gardon J, Boussinesq M, Kamgno J, Gardon-Wendel N, Duke BOL. Effects of standard and high doses of ivermectin on adult worms of Onchocerca volvulus: a randomised controlled trial. Lancet. 2002;360(9328):203-10.

19. Goa KL, McTavish D, Clissold SP. Ivermectin: A review of its antifilarial activity, pharmacokinetic properties and clinical efficacy in onchocerciasis. Drugs. 1991;42:640-58.

20. Habbema JDF, Stolk WA, Veerman LJ, de Vlas SJ. A rapid health impact assessment of APOC: technical report. 2007.

21. Osei-Atweneboana MY, Eng JKL, Boakye DA, Gyapong JO, Prichard RK. Prevalence and intensity of Onchocerca volvulus infection and efficacy of ivermectin in endemic communities in Ghana: a two-phase epidemiological study. Lancet. 2007;369(9578):2021-9.

22. Plaisier AP, Alley ES, Boatin BA, Oortmarssen GJv, Remme H, Vlas SJd, et al. Irreversible Effects of Ivermectin on Adult Parasites in Onchocerciasis Patients in the Onchocerciasis Control Programme in West Africa. J Infect Dis. 1995;172(1):204-10.

23. Turner HC, Walker M, Churcher TS, Basanez MG. Modelling the impact of ivermectin on River Blindness and its burden of morbidity and mortality in African Savannah: EpiOncho projections. Parasit Vectors. 2014;7:241.
